# Progestogen use and the risk of intracranial meningioma: a systematic review and meta-analysis

**DOI:** 10.1016/j.eclinm.2026.103791

**Published:** 2026-02-12

**Authors:** Benoit Hudelist, Alexandre Roux, Emmanuelle Huet-Mignaton, Isabelle Dufaure-Gare, Alessandro Moiraghi, Angela Elia, Maimiti Seneca, Corentin Provost, Joseph Benzakoun, Alexandre Gehanno, Catherine Oppenheim, Marc Zanello, Johan Pallud

**Affiliations:** a Service de Neurochirurgie, GHU-Paris Psychiatrie et Neurosciences, Site Sainte Anne, F-75014, Paris, France; b Université Paris Cité, Institute of Psychiatry and Neuroscience of Paris (IPNP), INSERM U1266, IMA-Brain, F-75014, Paris, France; c AMAVEA, France; d BIOSTATS, GHU-Paris Psychiatrie et Neurosciences, Site Sainte Anne, F-75014, Paris, France; e Service de Neuroradiologie, GHU-Paris Psychiatrie et Neurosciences, Site Sainte Anne, F-75014, Paris, France

**Keywords:** Pregnanes, Meningioma, Adverse effect, Neurosurgery, Medroxyprogesterone

## Abstract

**Background:**

Meningiomas are the most common primary brain tumours in adults. Concerns have emerged about a possible link between progestogen use and intracranial meningioma; we assessed this association.

**Methods:**

In this systematic review and meta-analysis, we searched PubMed/MEDLINE, Embase, Cochrane Library, EPI-PHARE database (from inception up to November 01, 2025), pharmacovigilance reports, and backward snowballing. Eligible publications were English or French epidemiological studies, reporting associations between progestogens and intracranial meningiomas. We excluded non-original reports, abstracts-only, and studies without eligible progestogen exposure or meningioma outcomes. We extracted summary data from published reports. Risk of bias was assessed with the Newcastle-Ottawa Scale, and certainty of evidence with GRADE. The primary outcome was intracranial meningioma. Secondary outcomes were malignancy, location, and regression. Random-effects models were used, and heterogeneity was assessed with I^2^; a narrative synthesis was also performed.

**Findings:**

Of 542 records screened, 78 studies were included in the review, and 14 high-quality observational studies in meta-analysis; all 14 were NOS high quality, although residual confounding and potential outcome misclassification cannot be excluded. Cyproterone acetate (CPA) was associated with increased meningioma risk (5 studies; 1047 exposed; pooled-OR 12.36 (95% CI: 7.47–20.45); I^2^: 73.8%; GRADE: moderate). Depot medroxyprogesterone acetate was also associated (6 studies; 842 exposed; pooled-OR 2.68 (95% CI: 1.72–4.19); I^2^: 92.7%; GRADE: low). Chlormadinone acetate (CMA), nomegestrol acetate (NOMAC), promegestone, medrogestone, and desogestrel showed signals of increased risk (CMA 3 studies, 164–683 exposed; NOMAC 3, 171–969; promegestone 1, 83; medrogestone 1, 42; desogestrel 2, 115–287). We did not pool these estimates due to sparse, heterogeneous evidence. No signal was found for norgestrel, levonorgestrel, progesterone, dydrogesterone, or spironolactone; evidence for dienogest and hydroxyprogesterone was insufficient. Regression after withdrawal was reported for CPA and NOMAC. Tumours were predominantly anterior/middle skull base, and malignant meningiomas were more frequent with CPA, CMA, and NOMAC.

**Interpretation:**

The certainty of evidence was limited by the observational design, residual confounding, heterogeneity, and imprecision for some exposures. Use of specific progestogens, particularly high dose macroprogestogens may be associated with an increased risk of intracranial meningioma. Transparent patient information and careful clinical and, where appropriate, imaging follow-up are essential.

**Funding:**

None.

**Research in context**

**Evidence before this study**

Before undertaking this study, we searched PubMed/MEDLINE, Embase, and the Cochrane Database of Systematic Reviews from database inception to November 01, 2025 (no language restrictions) to identify prior systematic reviews and meta-analyses on systemic progestogens (progestins) and intracranial meningioma risk. We also screened reference lists of eligible reviews and key pharmacovigilance/regulatory documents (e.g., EMA, ANSM/EPI-PHARE). Search terms combined “meningioma” with “progestogen/progestin” and drug-specific keywords (e.g., cyproterone acetate, chlormadinone acetate, nomegestrol acetate, medroxyprogesterone acetate). We included evidence syntheses with explicit eligibility criteria and a structured risk-of-bias appraisal, and excluded narrative reviews and case reports/series. We identified one prior systematic review/meta-analysis focused on cyproterone acetate, reporting an imprecise pooled estimate (RR: 3.78, 95% CI: 0.31–46.39) and judged at moderate-to-high risk of bias; certainty was low. No prior synthesis comprehensively evaluated other progestogens or outcomes such as tumour regression, malignant risk, or skull-base predilection.

**Added value of this study**

This systematic review and meta-analysis synthesise the largest contemporary evidence base across macro- and microprogestogens in diverse populations. It identifies specific agents associated with increased meningioma risk and supports a class effect for macroprogestogens. It also highlights desogestrel as a microprogestogen associated with meningioma during prolonged use. Beyond incidence, we document consistent radiological regression or stabilisation after drug withdrawal, quantify the elevated risk of malignant (WHO grade 3) meningioma for selected agents, and delineate a predilection for anterior and middle skull-base locations-findings directly relevant to screening and management. Overall, this study consolidates current knowledge and provides a robust foundation to guide clinical practice, policy, and future research.

**Implications of all the available evidence**

Given widespread, often long-term use of progestogens for contraception, endometriosis, menopausal therapy, and androgen suppression, clinicians should systematically document progestogen exposure in patients with known or suspected meningioma, consider withdrawal of high-risk agents when feasible, and consider MRI follow-up according to national recommendations and individual risk profiles, particularly in women with prolonged high-dose macroprogestogen exposure or with known/suspected meningioma. However, interpretation should consider key limitations: most available data are observational, exposure definitions and dose thresholds vary across studies, heterogeneity is substantial for some pooled estimates, and residual confounding and surveillance (detection) bias may inflate risk estimates, particularly in settings with structured MRI screening. Evidence for several non-CPA agents remains limited and imprecise, and generalisability to men and transgender populations is uncertain due to incomplete sex/gender reporting. Prescribers and regulators should integrate agent-specific risk profiles within an overall class-effect framework for macroprogestogens, reassess guidance for microprogestogens as higher-quality comparative studies become available.

## Introduction

Meningiomas are the most common primary tumours of the central nervous system in adults, accounting for around 40% of cases, with an incidence of 9.5 per 100,000 person-years.^1^^,^^2^ Most meningiomas are benign and develop slowly. They can be detected incidentally or present with neurological symptoms such as seizures, focal deficits, neurocognitive disturbances, or headaches.^3^ Their occurrence is higher in women with a post-pubertal sex ratio of 2:1, rising to 3:1 during peak reproductive years.^4^ Progesterone plays a significant role in the pathophysiology of meningiomas: an increase in meningioma size has been reported during pregnancy due to elevated progesterone levels,^5^ and studies have documented a 38–88% prevalence of progesterone receptors in meningioma cells.^6^, ^7^, ^8^ Reports of intracranial meningiomas in patients treated with high dose cyproterone acetate (CPA) and other potent progestogens have raised concerns about a potential causal link and prompted specifically designed epidemiological studies. Progestogens have been prescribed worldwide both for labelled indications (e.g., contraception, endometriosis and transgender hormone therapy) and off-label uses (e.g., acne and female alopecia) (Supplement data 1). Depot medroxyprogesterone acetate (DMPA) is among the most widely used injectable contraceptives globally, particularly in large-scale family planning programmes, resulting in prolonged exposure in millions of women. Large-scale cohort studies have explored the association between progestogens exposure and meningioma risk, yielding significant data with socioeconomic implications, given the widespread use of progestogens. Because meningiomas are slowly growing tumours, any causal effect of progestogens would be expected to manifest primarily after long-term or high cumulative exposure, whereas short-term courses are unlikely to materially affect risk. This systematic review and meta-analysis summarise current knowledge on the relationships between meningiomas and progestogens, with a particular focus on clinical implications, with specific attention to dose, route, duration of exposure, and clinical implications for patient management.

## Methods

### Search strategy and selection criteria

This systematic review was conducted and reported in accordance with the MOOSE guidelines^9^ and the PRISMA statement,^10^ following recent methodological recommendations for neurosurgical meta-analyses.^11^, ^12^, ^13^ The study was reviewed and approved by an institutional review board (IRB00011687, IRB #1: 2024/53).

The EPPI Reviewer software (v.6.15)^14^ was used for study screening and selection. From database inception up to November 01, 2025, we searched in the US National Library of Medicine (PubMed/MEDLINE), Embase (OVID), the Cochrane Library, and in the EPI-PHARE database identify all epidemiological studies examining associations between intracranial meningiomas and systemic progestogen exposure. Duplicate records were identified and removed. Study titles and abstracts were screened against the inclusion criteria. Full-text studies were imported and assessed for inclusion (see Supplement data 2). Additionally, we employed backward snowballing, and reviewed pharmacovigilance reports and national/international regulatory guidelines (including EMA and national agencies). The first author (B.H.) screened titles/abstracts; the senior author (J.P.) reviewed full texts and validated abstract-based exclusions; disagreements were resolved by consensus or, if needed, by a third reviewer (A.R.).

Publications were eligible if they met the following criteria: (1) full-text articles written in English or French; (2) reported an association between exposure to progestogens and meningiomas; (3) intracranial location of the meningioma. Systemic progestogens administered orally, by depot injection, or via intrauterine delivery systems were eligible; purely local or topical preparations without meaningful systemic absorption were excluded. Only epidemiological studies (cross-sectional, case–control, cohort) using data-driven methods were included in the meta-analysis. Descriptive cross-sectional reports, pharmacovigilance case series, and experimental studies were summarised narratively when relevant but were not pooled.

Using a standardised form, we extracted data for study characteristics (country, year, design, and number of cases), sex (as reported in the original articles), imaging characteristics (tumour volume, evolution over time, location). For progestogen we collected the characteristics (type, dose, and duration, and risk of meningiomas) and the route of administration (oral, injectable, intrauterine).

We used the Newcastle-Ottawa Scale (NOS) to evaluate methodological quality of non-randomised studies.^15^ This scale assesses quality across three domains: (1) study selection (4 points), (2) group comparability (2 points), and (3) exposure or outcome ascertainment (3 points). Studies scoring 7 points or more were classified as high quality. Two reviewers (B.H. and J.P.) independently applied the NOS and resolved discrepancies by discussion with a senior author (A.R.) when needed. Certainty of evidence was judged with the Grading of Recommendations Assessment, Development and Evaluation (GRADE) approach.^16^ GRADE assessments were performed at the level of each molecule-exposure contrast that entered quantitative synthesis, starting from “low” certainty for observational data and downgrading for risk of bias, inconsistency, indirectness, imprecision, or suspected publication bias as appropriate; for exposures with large, consistent effects and a plausible dose–response gradient, we considered upgrading by one level. When multiple studies assessed similar exposures based on potentially overlapping populations and timeframes, we prioritised cohort studies over case–control designs, favouring those with larger sample sizes and more robust dose–response analyses. This strategy aimed to minimise the risk of double-counting individuals and to preserve the independence of effect estimates included in the meta-analysis.

### Data analysis

We performed random-effects meta-analyses on the log-relative risk scale using generic inverse-variance weighting and a restricted maximum likelihood estimator for τ^2^. Effect estimates were log-transformed before pooling and back-transformed for interpretation. Heterogeneity was summarised by I^2^ and Tau.^2^ Forest plots display pooled relative risks with 95% CI. We interpreted I^2^ as low (<30%), moderate (30–60%), substantial (60–90%) and considerable (>90%). When heterogeneity was substantial, we also reported 95% prediction intervals and performed leave-one-out influence analyses (see Supplement data 5). Quantitative synthesis was restricted to molecule-specific contrasts with at least two comparable studies. All analyses used R (v4.0.5) and the meta and metafor packages^17^ (Precision in Supplement data 6).

We prespecified the OR as the pooling metric and performed meta-analyses on the log-OR scale using the generic inverse-variance method. When only RR or HR were available, we used the reported adjusted estimates on the log scale and treated them as approximations of ORs, given the low absolute incidence of intracranial meningioma (precision is provided in Supplement data 7).

### Role of the funding source

There was no funding source for this study. The authors had full access to all the data included in the study. BH and JP had final responsibility for the decision to submit for publication.

## Results

**Fig. 1:**
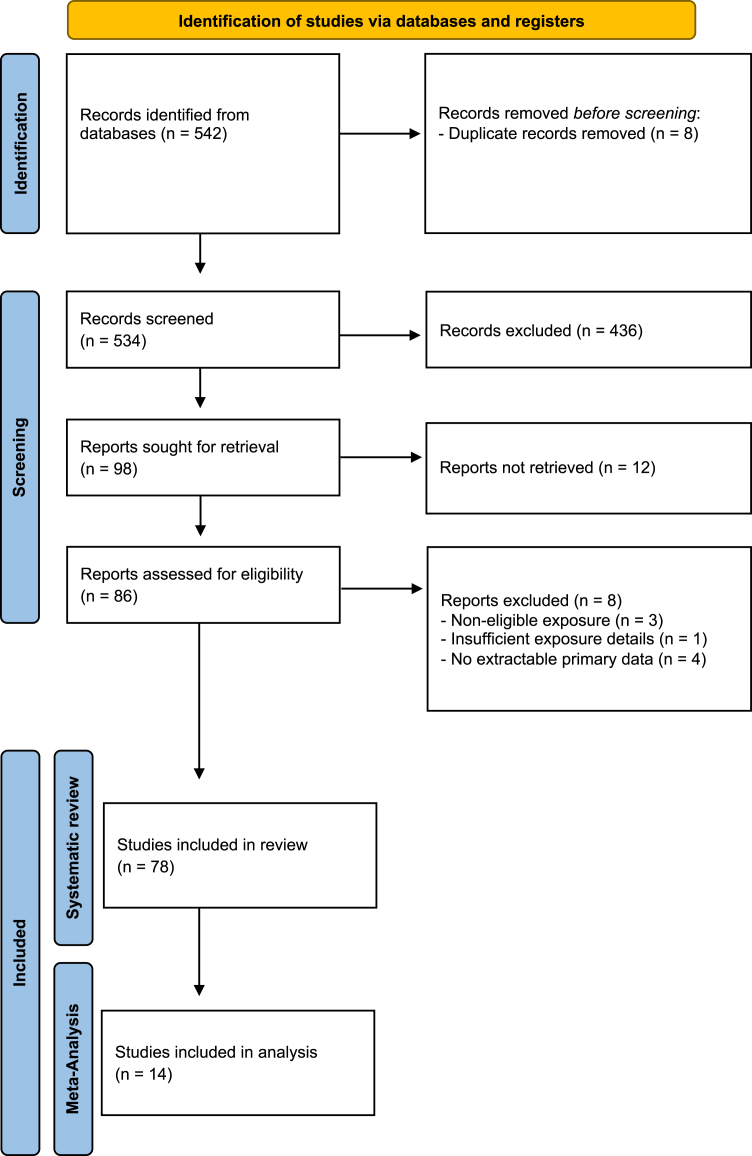
Study Flowchart.

The PRISMA flow diagram is presented in Fig. 1. 542 studies were initially identified: eight duplicates were withdrawn, 436 were excluded based on title and abstract, 12 were not retrieved, and eight were excluded based on full text. Finally, 78 studies were included in final review. Among these, 14 studies were deemed eligible for inclusion in the meta-analysis (presented in Table 1), 5 were cohort studies and 9 were case–control studies. All 14 studies were classified as high quality according to the NOS scoring system (Supplement data 8); however, even studies rated as “high quality” on the NOS remained subject to residual confounding and, in some registry-based analyses, potential outcome misclassification. According to GRADE, the certainty of evidence was moderate for high-dose CPA and low for DMPA, and low to very low for other progestogens (reflecting the observational design, residual confounding, heterogeneity, and imprecision for several exposures) (Supplement data 9). Included articles are listed in Supplement data 10, 11 and 12.

**Table 1:** Characteristics of included studies.

Study (first author, year)	Country	Design	Study period	Exposure	Comparator	Sample size	Adjusted effect (OR/RR/HR) (95% CI)
Gil et al. (2011)	Spain; BIFAP primary care database	Population-based cohort	2001–2007	High-dose CPA (≥50 mg/day)	Non-exposed	Exposed: 2474 Control: 2,112,479	RR: 11.4 (95% CI: 4.3–30.8)
Cea-Soriano et al. (2012)	UK; THIN primary care database	Case-control	1996–2008	High-dose CPA (≥50 mg/day)	Non-users of the drug	Cases: 745 Controls: 10,000	OR: 6.30 (95% CI: 1.37–28.94)
Weill et al. (2021)	France; SNDS (national health data system)	Population-based cohort	2007–2014	CPA (cumulative dose ≥3 g within the first 6 months)	Slightly exposed (≤3 g within first 6 months)	Exposed: 139,222 Control: 114,555	HR: 6.6 (95% CI: 4.0–11.1)
Mikkelsen et al. (2022)	Denmark; nationwide registers	Population-based cohort	1995–2017	High-dose CPA (cumulative dose ≥10 g in total)	Non-exposed	Exposed: 781 Control: 5,728,672	HR: 19.2 (95% CI: 10.3–35.8)
Hoisnard et al. (2022)	France; SNDS (national health data system)	Case-control	2009–2018	High-dose CPA (≥25 mg/day) NOMAC (3.75–5 mg/day) CMA (2–10 mg/day)	Non-exposed	Cases: 25,216 Controls: 126,080	CPA: OR: 18.3 (95% CI: 16.0–21.1) CMA: OR: 4.7 (95% CI: 4.5–5.3) NOMAC: OR: 4.7 (95% CI: 4.3–5.1)
Nguyen et al. (2024)	France; SNDS (national health data system)	Population-based cohort	2007–2017	NOMAC (cumulative dose ≥150 mg within the first 6 months)	Slightly exposed (≤150 mg within first 6 months)	Exposed: 535,115 Control: 525,664	RR: 2.9 (95% CI: 2.4–3.7)
Roland et al. (2024)	France; SNDS (national health data system)	Population-based cohort	2007–2017	CMA (cumulative dose ≥360 mg within the first 6 months)	Slightly exposed (≤360 mg within first 6 months)	Exposed: 469,976 Control: 358,523	RR: 3.1 (95% CI: 2.4–4.0)
Roland et al. (2024)	France; SNDS (national health data system)	Case-control	2009–2018	DMPA (150 mg) CPA (50, 100 mg) CMA (5, 10 mg) NOMAC (3.75, 5 mg) Promegestone (0.125, 0.25, or 0.5 mg) Medrogestone (5 mg)	Non-exposed	Cases: 18,061 Controls: 90,305	DMPA: OR: 5.55 (95% CI: 2.27–13.56) CPA: OR: 19.2 (95% CI: 16.61–22.22) CMA: OR: 3.87 (95% CI: 3.48–4.30) NOMAC: OR: 4.93 (95% CI: 4.50–5.41) Promegestone: OR: 2.39 (95% CI: 1.85–3.09) Medrogestone: OR: 3.49 (95% CI: 2.38–5.10)
Griffin et al. (2024)	USA; IBM MarketScan register	Case-control	2006–2022	DMPA (150 mg IM or 104 mg SC)	Non-users of the drug	Cases: 117,503 Controls: 1,072,907	OR: 1.68 (95% CI: 1.50–1.87)
EPI-PHARE et al. (2024)	France; SNDS (national health data system)	Case-control	2020–2023	Desogestrel (75 μg, exposure in the previous year)	Non-users of the drug	Cases: 8391 Controls: 83,910	OR: 1.25 (95% CI: 1.10–1.42)
Xiao et al. (2025)	USA; TriNetX register	Population-based cohort	2004–2024	DMPA (150 mg)	Non-exposed	Exposed: 88,667 Controls: 88,667	RR: 2.43 (95% CI: 1.77–3.33)
Tettamanti et al. (2025)	Sweden; Swedish Cancer Register + Prescribed Drug Register	Case-control	2007–2015	DMPA (150 mg)	Non-users of the drug	Cases: 1055 Controls: 21,100	OR: 5.49 (95% CI: 4.51–6.67)
Reynolds et al. (2025)	USA (Alabama); Medicaid claims database	Case-control	2010–2023	DMPA (150 mg)	Non-users of the drug	Cases: 469 Controls: 4690	OR: 1.81 (95% CI: 1.14–2.89)
Griffin et al. (2025)	USA; University of Alabama at Birmingham (single centre)	Case-control	2015–2024	DMPA (150 mg)	Non-users of the drug	Cases: 241 Controls: 723	OR: 1.9 (95% CI: 0.99–4.5)

CPA: Cyproterone acetate; NOMAC: Nomegestrol Acetate; CMA: Chlormadinone acetate; DMPA: Depot medroxyprogesterone acetate.

The classification of synthetic progestogens, their modes of action, indications, and dosing regimens is summarised in Table 2 and Supplement data 3; a more detailed narrative description is provided in Supplement data 4.

**Table 2:** Summary of evidence on intracranial meningioma risk associated with progestogens.

∗Use restricted/monitored in several countries due to meningioma risk (class-/molecule-specific).

†Contexts vary by country and may be off-label.

%Presented in pooled OR (CPA and DMPA) or OR.

∗∗GRADE certainty ratings were assigned for exposures with sufficient comparative epidemiological evidence; for other exposures, evidence was insufficient to support a GRADE assessment.

HRT: Hormone Replacement Therapy; PMS: Premenstrual Syndrome; AUB: Abnormal uterine bleeding.

### Meningioma risk

#### Cyproterone acetate (CPA)

CPA is the first progestogen identified as having a potential association with meningiomas in 2007.^18^ Since, case reports and cohort studies involving both men and women have documented the occurrence of meningiomas in the setting of CPA exposure.^18^, ^19^, ^20^, ^21^, ^22^, ^23^, ^24^, ^25^, ^26^, ^27^, ^28^, ^29^, ^30^, ^31^, ^32^, ^33^, ^34^, ^35^, ^36^, ^37^, ^38^, ^39^, ^40^, ^41^, ^42^, ^43^, ^44^, ^45^, ^46^, ^47^, ^48^, ^49^, ^50^, ^51^, ^52^, ^53^, ^54^ All studies converge on a clear association between high-dose or high cumulative CPA. Gil et al. in 2011^55^ found an increased risk of meningioma in high-dose individuals using CPA (aRR: 11.4, 95% CI: 4.3–30.8), with 4 cases among 2474 individuals exposed to CPA versus individuals not exposed to CPA. Cea-Soriano et al. in 2012^56^ identified an increased risk of meningioma associated with high-dose CPA (OR: 6.30, 95% CI: 1.4–28.9; 3 cases) but not with low-dose CPA. High-dose CPA exposure occurred almost exclusively in men, whereas low-dose CPA in combined oral contraceptives concerned women. A large French cohort study by Weill et al. in 2021^57^^,^^58^ identified a risk of meningioma in patients exposed to CPA (HR: 6.6, 95% CI: 4.0–11.1; 69 cases). They observed a dose–response relationship with a higher risk for high cumulative dose of CPA (>60 g; HR: 21.7, 95% CI: 10.8–43.5; 15 cases). Notably, after one year of discontinuing CPA, the risk of meningioma remained higher in the exposed group than in the non-exposed group (HR: 1.8, 95% CI: 1.0–3.2; 34 cases). A subsequent French study by Hoisnard et al. in 2022,^59^ confirmed the risk of meningioma by CPA exposure (OR: 18.3, 16.0–21.1; 961 cases). They observed the strongest association with CPA exposure >1 year (OR: 22.7, 95% CI: 19.5–26.6; 931 cases). A Danish cohort study by Mikkelsen et al.^60^ in 2022 confirmed the dose-dependent association between CPA exposure and meningioma risk (cumulative CPA exposure of 0.1–10 g, HR: 7.0, 95% CI: 3.1–15.6, 6 cases/cumulative exposure >10 g, HR: 19.2, 95% CI: 10.3–35.8, 10 cases). A previous meta-analysis by Lee et al.^61^ attempted to pool progestogen-meningioma data but contained several numerical inaccuracies (e.g., misreported sample sizes in major registry studies and inconsistent handling of person-time) and reported a non-significant association between CPA use and meningioma risk (RR: 3.78, 95% CI: 0.31–46.39). Considering these methodological issues, we did not rely on their pooled estimates and instead present an independent synthesis based on a re-extraction and re-analysis of the original studies. Several research considered low dose of CPA only, and none of these found any association with meningioma, even with long-term use.^44^^,^^55^, ^56^, ^57^ The recent report of EPI-PHARE Scientific Interest Group of the French National Agency for Medicines and Health Products Safety by Roland et al. in 2023^62^^,^^63^ confirmed the significant association between CPA exposure and meningioma risk (OR: 19.21, 95% CI: 16.61–22.22; 891 cases).

**Fig. 2:**
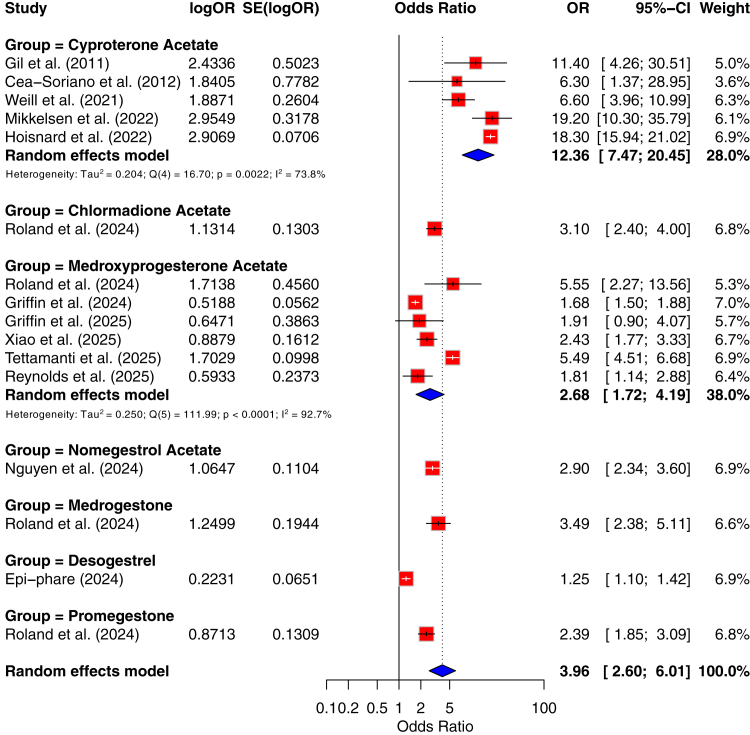
Risk of meningioma by progestogen use. Results of the meta-analysis.

After converting HR/RR to OR and restricting the analysis to high-dose or high cumulative CPA exposure, the pooled OR was 12.36 (95% CI: 7.47–20.45), with substantial heterogeneity (I^2^ = 73.8%, τ^2^ = 0.204; n = 5 studies, 1047 cases). Leave-one-out analyses did not change direction. Studies assessing the risk of meningioma by CPA use are summarised in Fig. 2 and Table 3 (Precision in Supplement data 13).

**Table 3:** Summary of pooled relative risks for progestogen exposures with formal meta-analyses.

Progestogen exposure	Exposure contrast	Number of studies	Exposed meningioma cases (events)	Pooled OR (95% CI)	Heterogeneity (I^2^)	Certainty of evidence (GRADE)
Cyproterone acetate (CPA), oral	High-dose or high cumulative CPA vs no CPA	5	1047^a^	12.36 (7.47–20.45)	73.8%	Moderate
Depot medroxyprogesterone acetate (DMPA), injectable	Any injectable DMPA use vs no DMPA/non-active comparators	6	842^b^	2.68 (1.72–4.19)	92.7%	Low

Participants providing data refers to the number of meningioma cases (events) among exposed participants contributing to the pooled estimate; total cohort sizes were not consistently reported across studies.

OR: Odds Ratio.

a 4 + 3 + 69 + 10 + 961.

b 9 + 480 + 7 + 131 + 186 + 29.

#### Chlormadinone acetate (CMA)

The potential association between CMA and meningiomas was first reported by Shimizu et al.^64^ in 2008. In 2020, Roux et al.^65^ reported another case of meningioma volume decrease following CMA withdrawal, further suggesting a possible link. In 2021–2022 Malaize et al.^39^ Graillon et al.^41^^,^^42^ Devalckeneer et al. and Samoyeau et al.^34^ reported meningioma stabilisation or regression after CMA withdrawal. Before 2022, Grandi et al.^66^ cautioned against drawing definitive conclusions due to the limited available data. An epidemiological study conducted by the EPI-PHARE Scientific Interest Group by Hoisnard et al.^59^ in 2022 identified a significant risk of meningiomas with CMA exposure (OR: 4.7, 95% CI: 4.5–5.3; 683 cases). An EPI-PHARE SNDS (French National Health Data System ou Système national des données de santé) cohort report in 2021 first identified an increased meningioma risk with prolonged high-dose CMA exposure, later confirmed in the peer-reviewed update by Roland et al. in 2024 (age-adjusted RR: 3.1, 95% CI: 2.4–4.0; 164 cases).^67^^,^^68^ In the most exposed (cumulative dose) group (>8.64 g) the age-adjusted RR of developing a meningioma was 6.9 (95% CI: 5.1–9.2; 86 cases) compared to the control group, suggesting a dose–response effect. The most recent report of the EPI-PHARE Scientific Interest Group confirmed the significant association between exposure of CMA and meningiomas risk (OR: 3.87, 95% CI: 3.48–4.30; 628 cases).^62^^,^^63^

Given the limited number and heterogeneity of designs, we did not perform a pooled meta-analysis for CMA and provide a narrative synthesis (summarised in Fig. 2, precision in Supplement data 13).

#### Medroxyprogesterone acetate (MPA)

The potential association between MPA and meningiomas was first assessed by Korhonen et al.^69^ in 2012 and did not identify an increased risk. Study limitations encompassed the inclusion of all progestogens in the same group and the restriction to only postmenopausal hormone replacement therapy exposure. Abou-Al-Shaar et al.^70^ in 2023 first suggested the association between MPA use and meningioma risk. Pourhadi et al.^71^ in 2023 conducted an epidemiological study in Denmark on postmenopausal hormone therapy and meningioma without specific analysis of MPA. They reported higher risk both in the oestrogen-progestogen group (HR: 1.21, 95% CI: 1.06–1.37; 423 cases) and in the progestogen-only group (HR: 1.28, 95% CI: 1.05–1.54; 143 cases), suggesting a potential association between progestin-containing menopausal hormone therapy, largely based on MPA in that setting, and meningioma. A case–control study based on the USA registry by Griffin et al.^72^ in 2024 suggested that oral MPA exposure was not associated with an increased risk of meningioma, whereas DMPA exposure was (OR: 1.68, 95% CI: 1.50–1.87; 480 cases), and that the risk increased with longer exposure duration (exposure ≤1 year, OR: 1.23, 95% CI: 1.10–1.38; 457 cases, and exposure >3 years, OR: 2.50, 95% CI: 2.06–3.04; 170 cases). The 2024 report of the EPI-PHARE Scientific Interest Group^68^^,^^69^ reported a significant association between DMPA exposure and meningiomas in France (OR: 5.55, 95% CI: 2.27–13.56; 9 cases).^62^^,^^63^ More recently, in 2025, several large population-based studies focusing specifically on DMPA have confirmed this signal: Xiao et al.^73^ found, in 2025, an increased risk of meningioma among individuals using DMPA (RR: 2.43, 95% CI: 1.77–3.33; 131 cases), Reynolds et al.^74^ reported in a nested case–control study an elevated risk with DMPA “ever-use” (OR: 1.81, 95% CI: 1.14–2.89; 29 cases) with markedly higher estimates for prolonged exposure and little or no excess risk for short-term use. Tettamanti et al.^75^ observed in a Swedish nationwide case–control study a strong association between DMPA and meningioma (OR: 5.49, 95% CI: 4.51–6.67; 186 cases). In addition, a more recent US case–control study, by Griffin et al.^76^ using active and non-active hormonal comparators reported a non-significant but directionally consistent excess risk for any prior DMPA exposure (OR: 1.91, 95% CI: 0.99–4.50; 7 cases), further supporting a possible class effect of DMPA. Overall, these convergent data indicate that the excess risk is largely driven by long-term or repeated exposure to DMPA, whereas oral MPA and short-term courses do not show a consistent association with meningioma.

The pooled OR, restricted to DMPA, was 2.68 (95% CI: 1.72–4.19), with substantial heterogeneity (I^2^ = 92.7%, τ^2^ = 0.25; n = 6 studies, 842 exposed cases). Leave-one-out analyses did not change direction. Studies assessing the risk of meningioma by MPA use are summarised in Fig. 2 and Table 3 (Precision in Supplement data 13). In contrast, studies assessing oral MPA, mainly as part of menopausal hormone therapy or oral progestin-only regimens, generally reported no or only modest increases in meningioma risk. Taken together, these results support that any potential risk with oral MPA is likely markedly lower than that observed with depot DMPA, and may be confined to long-term, continuous use in specific populations.

#### Nomegestrol acetate (NOMAC)

The potential association between NOMAC and meningioma was first reported by Gruber et al.^77^ in 2004. Gruber et al.^78^ in 2011 reported four patients, all of whom had meningiomas diagnosed while undergoing NOMAC treatment, and none showed signs of recurrence after surgery and NOMAC withdrawal. Seven additional case reports have described similar instances of meningioma in patients treated with NOMAC^20^^,^^25^^,^^79^, ^80^, ^81^, ^82^, ^83^ and numerous studies have been published on patients with meningioma exposed to NOMAC.^32^^,^^34^^,^^35^^,^^38^^,^^39^^,^^41^, ^42^, ^43^^,^^47^^,^^78^ Between 2021 and 2022, Samarut et al.^32^ Malaize et al.^39^ Graillon et al.^41^ Devalckeneer et al.^42^ and Samoyeau et al.^34^ suggested an association between NOMAC use and meningioma. However, the small sample sizes (ranging 1–12 patients) precluded establishing a definitive link. An epidemiological study conducted by the EPI-PHARE Scientific Interest Group^39^ identified a significant risk of developing a meningioma in patients exposed to NOMAC (OR: 4.7, 95% CI: 4.3–5.1; 969 cases).^59^ EPI-PHARE first reported an increased meningioma risk with prolonged/high-dose NOMAC exposure in a 2021 SNDS study, later confirmed and expanded in an updated analysis in 2024 (age-adjusted RR: 2.9, 95% CI: 2.4–3.7; 171 cases), with a marked dose–response gradient (RR 12.0 for >6 g cumulative exposure).^84^^,^^85^ The last report of the EPI-PHARE Scientific Interest Group published in 2024 reported a significant association between exposure to NOMAC and meningiomas (OR: 4.93, 95% CI: 4.50–5.41; 925 cases).^62^^,^^63^

Given the limited number and heterogeneity of designs, we did not perform a pooled meta-analysis and provide a narrative synthesis (summarised in Fig. 2, precision in Supplement data 13).

#### Promegestone

Abijaoude et al.^82^ in 2021 reported a single case of an osteomeningioma that developed during promegestone therapy. Apra et al.^35^ in 2020 and Graillon et al.^41^ in 2021 reported series where meningiomas were observed concomitantly with promegestone treatment, suggesting a potential association. The recent report of the EPI-PHARE Scientific Interest Group^69^^,^^86^ identified significant risk of developing a meningioma among patients exposed to promegestone (OR: 2.39, 95% CI: 1.85–3.09; 83 cases).^62^^,^^63^ Prolonged use of promegestone ≥1 year was associated with a higher risk (OR: 2.74, 95% CI: 2.04–3.67; 66 cases).

Given the limited number and heterogeneity of designs, we did not perform a pooled meta-analysis and provide a narrative synthesis (summarised in Fig. 2, precision in Supplement data 13).

#### Medrogestone

The potential association between medrogestone and meningiomas was first suggested by Apra et al.^35^ in 2020. The report of the EPI-PHARE Scientific Interest Group by Roland et al.^62^^,^^63^ identified significant risk of developing a meningioma among patients exposed to medrogestone (OR: 3.49, 95% CI: 2.38–5.10; 42 cases). Prolonged use of medrogestone ≥1 year was associated with a higher risk (OR: 4.08, 95% CI: 2.72–6.10; 40 cases).

Given the limited number and heterogeneity of designs, we did not perform a pooled meta-analysis and provide a narrative synthesis (summarised in Fig. 2, precision in Supplement data 13).

#### Desogestrel

The association between desogestrel and meningiomas was first explored in the recent report of the EPI-PHARE Scientific Interest Group.^87^ They identified significant risk of developing a meningioma among patients exposed to desogestrel (OR: 1.25, 95% CI: 1.10–1.42; 287 cases). The risk appears after five years of desogestrel use (OR: 1.70, 95% CI: 1.39–2.08; 115 cases) and increases with duration of desogestrel exposure (5–6 years of use, OR: 1.51, 95% CI: 1.17–1.94; 71 cases, and ≥7 years of use, OR: 2.09, 95% CI: 1.51–2.90, 44 cases). If desogestrel has been stopped >1 year, the risk of developing a meningioma disappeared (OR: 0.83, 95% CI: 0.63–1.09; 58 cases). Building on this evidence, Roland et al.^88^ published in 2025 a large national case–control study, based on the same cohort, and demonstrated that prolonged use (≥5 years) of desogestrel 75 μg was associated with an increased risk of intracranial meningioma (OR: 1.70, 95% CI: 1.39–2.08, 115 cases), with a stronger association for use ≥7 years (OR: 2.09, 95% CI: 1.51–2.90, 44 cases).

Given the limited number and heterogeneity of designs, we did not perform a pooled meta-analysis and provide a narrative synthesis (summarised in Fig. 2, precision in Supplement data 13).

#### Other progestogens

Several studies explored the association between other progestogens (including dydrogesterone, levonorgestrel, norethisterone, progesterone, hydroxyprogesterone, spironolactone and dienogest) and meningiomas. None of them identified a clear link.^35^^,^^41^^,^^44^^,^^69^^,^^71^^,^^86^^,^^88^, ^89^, ^90^ The EPI-PHARE Scientific Interest Group investigated the use of progestogens widely prescribed in France between 2009 and 2018.^62^ They found no significant association for an increased risk of intracranial meningioma surgery and exposure to oral or intravaginal progesterone (OR: 0.88 (95% CI: 0.78–0.99); 329 cases), percutaneous progesterone (OR: 1.11 (95% CI: 0.89–1.40); 90 cases), dydrogesterone (OR: 0.96 (95% CI: 0.81–1.14); 156 cases), spironolactone (OR: 0.95 (95% CI: 0.84–1.09); 264 cases), dienogest (OR: 1.48 (95% CI: 0.41–5.35); 3 cases), levonorgestrel 52 mg intrauterine system (OR: 0.94 (95% CI: 0.86–1.04); 566 cases), and levonorgestrel 13.5 mg intrauterine system (OR: 1.39 (95% CI: 0.70–2.77); 10 cases). No exposed cases were found for hydroxyprogesterone. However, the EPI-PHARE Scientific Interest Group underlined that no conclusions could be drawn regarding dienogest or hydroxyprogesterone due to the small number of people receiving these drugs.

**Fig. 3:**
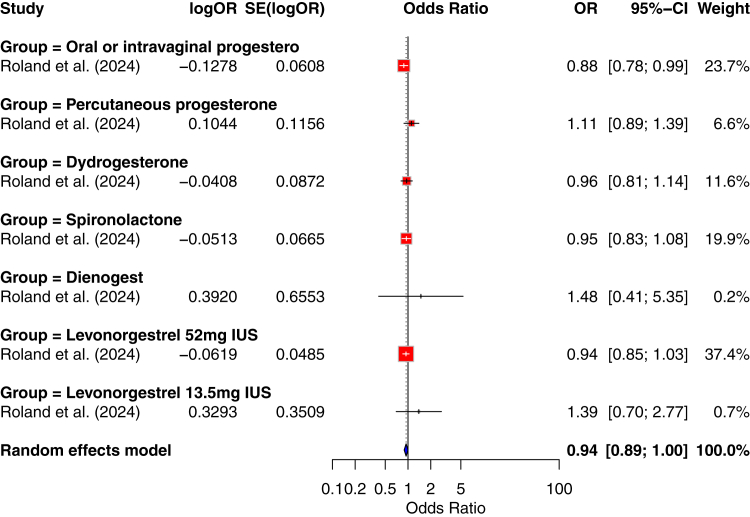
Progestogens not associated with a higher risk of meningiomas.

Given the limited number and heterogeneity of designs, we did not perform a pooled meta-analysis and provide a narrative synthesis (summarised in Fig. 3).

### Malignant meningioma risk

Malignant WHO grade 3 meningiomas are rare, ranging 1–2% of cases.^1^ As reported by Roux et al.^65^ in 2022, cases of malignant meningiomas have been observed in association with progestogens, suggesting a potentially higher risk linked to these medications. The EPI-PHARE Scientific Interest Group^39^ demonstrated that progestogen exposure was associated with a significantly increased risk of malignant meningiomas in women for CPA (OR: 23.7, 95% CI: 8.1–69.8; 20 cases), NOMAC (OR: 4.9, 95% CI: 2.2–10.8; 13 cases), and CMA (OR: 2.8, 95% CI: 1.3–5.9; 11 cases).^59^ These findings were confirmed by Roland et al. in 2024 who reported a significant increased risk of malignant meningiomas for CPA (OR: 22.5, 95% CI: 7.61–66.48; 18 cases), NOMAC (OR: 4.95, 95% CI: 2.29–10.68; 13 cases), and CMA (OR: 5.78, 95% CI: 2.39–14.00; 11 cases).^62^ No malignant meningioma was observed in cases exposed to medrogestone, medroxyprogesterone acetate, or promegestone.

### Meningioma growth

The effects of drug withdrawal on the spontaneous growth of untreated meningiomas have been reported in 16 studies for CPA^19^, ^20^, ^21^, ^22^, ^23^, ^24^^,^^31^^,^^32^^,^^34^^,^^37^, ^38^, ^39^, ^40^, ^41^, ^42^, ^43^, ^44^ (ranging from 1–188 cases), in 10 studies for NOMAC^20^^,^^34^^,^^38^^,^^39^^,^^41^, ^42^, ^43^^,^^77^^,^^78^^,^^81^ (ranging from 1 to 6 cases), in seven studies for CMA^34^^,^^38^^,^^39^^,^^41^, ^42^, ^43^^,^^64^ (one case per study), in one study for MPA^70^ (10 cases), and in two studies for promegestone^41^^,^^82^ (one case per study). No study has been identified for other progestogens. For CPA, treatment discontinuation leads to significant tumour shrinkage on MRI: regression in 36–92%,^37^ stability in 8–85.2%,^39^ and continued growth in 0–29%.^42^ Most studies included various progestogens and examined volume variations across all progestogen-exposed patients, making difficult to draw conclusions for each one^34^^,^^39^^,^^43^: regression in 0–12.5%, stability in 60.0–62.5%, and continued growth in 25.0–40.0% for NOMAC^38^; no regression, stability in 66.7%, and continued growth in 33.3% for CMA.^38^ Graillon et al.^41^ combined CMA and NOMAC data (11 cases total) and found regression in 18%, stability in 64%, and continued growth in 18%.

Abou-Al-Shaar et al.^70^ followed 10 women after MPA withdrawal: in five, tumours shrank; in three, DMPA had been discontinued less than one year earlier, precluding firm conclusions; two were lost to follow-up. Graillon et al.^41^ and Abi Jaoude et al.^82^ each reported a single case of meningioma regression following promegestone withdrawal. An illustration is shown in Supplement data 15.

The evolution of osteomeningiomas after progestogen withdrawal remains unclear. Reports suggest that the dural component of meningiomas regressed and that the osseous component continued to grow and even exhibited an increased growth rate following progestogen withdrawal.^31^^,^^43^^,^^82^

### Tumour location

**Fig. 4:**
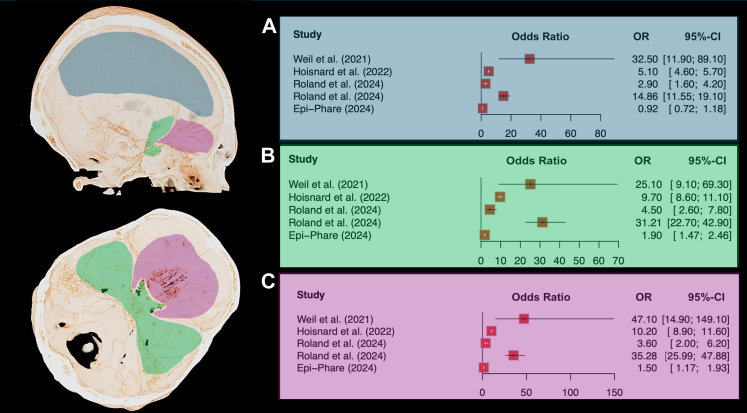
Tumour location. Tumour location, regardless of the progestogen, is classified as convexity (A.), middle skull base (B.), and anterior skull base (C.), represented in blue, green, and purple, respectively.

Published series reported a predominant meningioma location at the anterior and middle skull bases, regardless of the progestogen used^18^, ^19^, ^20^, ^21^, ^22^, ^23^, ^24^, ^25^, ^26^, ^27^, ^28^, ^29^^,^^31^^,^^32^^,^^34^^,^^37^, ^38^, ^39^, ^40^, ^41^, ^42^, ^43^, ^44^^,^^46^^,^^47^^,^^53^^,^^54^^,^^64^^,^^65^^,^^70^^,^^77^, ^78^, ^79^, ^80^, ^81^, ^82^, ^83^^,^^86^ as summarised in Fig. 4 and Supplement data 16. This contrasts with the predominant cranial convexity location for meningioma occurrence in the general population.^91^ Across the seven largest epidemiological studies,^57^^,^^59^^,^^62^^,^^67^^,^^84^^,^^87^^,^^92^ regardless of the specific progestogen examined, the anterior skull base ranged 20–37% and the middle skull base ranged 20–39%. In contrast, despite cranial convexity being the most common site in the general population,^91^ none of these studies reported convexity location rate surpassing those of the anterior or middle skull bases.

## Discussion

This systematic review and meta-analysis investigated the link between progestogen use and the risk of meningioma and included 14 studies in the final analysis. We show: (1) an increased risk of meningioma for high-dose CPA and DMPA, and lower-certainty signals suggesting increased risk for other macroprogestogens (CMA, NOMAC, promegestone, medrogestone) and for one microprogestogen (desogestrel); (2) no significant risk of meningioma with norgestrel and levonorgestrel; (3) no conclusion regarding dienogest or hydroxyprogesterone due to the small number of exposed people; (4) an increased risk of malignant meningioma with CPA, CMA, and NOMAC; (5) drug withdrawal seemed to lead to a decrease in meningioma growth (shrinkage or stability of the tumour in the majority of reported cases); (6) a predominant location at the anterior and middle skull bases.

Our conclusions are explicitly calibrated to the certainty of evidence (GRADE): for CPA, moderate-certainty evidence supports a strong, precautionary recommendation, whereas low-certainty evidence for DMPA warrants cautious, conditional statements and emphasises the need for replication and long-term follow-up. For other progestogens, certainty ranges from low to very low, and effect sizes should be interpreted primarily as signals rather than precise quantifications of risk.

Due to the widespread use of CPA in France (55,000 individuals’ exposure in 2019, around 400,000 between 2006 and 2015),^57^ the association between progestogen exposure and meningioma risk is a major public-health and regulatory issue. In 2018, the French health insurance agency conducted a large cohort analysis that revealed a strong dose-dependent association between CPA use and intracranial meningioma surgery.^44^ In September and October 2018, French health authorities implemented measures to mitigate this risk^93^; prescribers received recommendations to regularly assess the benefit-to-risk balance of CPA use and to prescribe brain MRI at treatment initiation, five years later, and then every two years in cases of treatment continuation. In June 2019, the French health insurance agency listed treated patients to each CPA prescribers and contacted patients who had received reimbursements for CPA to inform them of the risk of meningioma and counsel them to consult their practitioners. Since July 2019, an annual information form, co-signed by the patient and the prescriber, has also been required for CPA dispensing. In 2025, the EPI-PHARE Scientific Interest Group evaluated the impact of measures implemented in France in 2018 and 2019 and found that the number of individuals exposed to CPA decreased by 85% (from 55,000 in August 2018 to 7900 in December 2021).^94^ Furthermore, the annual rate of meningioma surgeries linked to CPA exposure decreased by about 93% over the same period.^94^ In France, other progestogens were progressively integrated into the MRI monitoring process: NOMAC and CMA in July 2021,^95^ and MPA and medrogestone in July 2024.^96^ Notifications were initially sent to prescribers, followed by communications to exposed patients. Furthermore, each treatment was incorporated into a screening algorithm, and an information form now requires joint signatures from both patients and prescribers. As of 2025, several high-dose macroprogestogen formulations investigated in this review are being withdrawn or not renewed in Europe, including CPA ≥50 mg, CMA 5–10 mg, higher-dose NOMAC (3.75–5 mg), oral promegestone, and 5 mg medrogestone tablets. Beyond France, the EMA and several national agencies have issued multiple safety opinions and Dear Healthcare Professional Communications restricting indications, limiting doses, or withdrawing specific high dose macroprogestogen formulations (including CPA, CMA, NOMAC, promegestone and medrogestone) in 2024–2025. These regulatory decisions explicitly acknowledge the epidemiological signals summarised in our review, but they go beyond the direct evidence from individual studies by adopting a precautionary, population-level stance. In contrast, regulatory action in other regions, particularly in the USA, has so far been more limited: labelling has been updated to mention meningioma as a potential adverse effect, yet no coordinated restriction comparable to EMA decisions has been implemented despite extensive multidistrict litigation and numerous civil lawsuits alleging progestogen-associated meningiomas. This divergence illustrates how the same body of observational evidence can lead to markedly different regulatory responses depending on legal frameworks, health-system priorities, and tolerance for precautionary intervention.

Since the seven most used macroprogestogens increase the risk of developing a meningioma in the current literature, a class effect is plausible and should be considered, although it cannot be demonstrated formally, and the magnitude of risk clearly differs between molecules and routes of administration. Progestogen-associated meningiomas tend to exhibit a higher frequency of somatic PIK3CA mutations, indicating a hormone-driven mutational shift that promotes growth and enhances cell invasion.^47^^,^^97^ In contrast, several studies have explored microprogestogens without evidence of an association with meningiomas. The EPI-PHARE Scientific Interest Group recently demonstrated that desogestrel is associated with an increased risk of meningiomas^87^ and, although the risk is low, it should be considered during prescription making process, particularly for patients with a known meningioma. Further studies should be conducted, and health insurance providers should be aware of the potential risks associated with microprogestogens. Across large registries and cohorts, the excess risk associated with macroprogestogens and DMPA was consistently concentrated in women with long-term or high cumulative exposure, whereas short-term or sporadic use rarely showed a clear signal, which is consistent with the long latency and slow growth of meningiomas.

Based on current knowledge, for patients with an intracranial meningioma, discontinuing macroprogestogens with established associations to meningiomas should be systematically considered and is generally recommended, taking into account indication and alternatives. While there is no conclusive evidence linking microprogestogens to meningioma development, treatment cessation and close monitoring must be balanced with the necessity of continuing the microprogestogen if a meningioma is diagnosed. Whenever possible, progestogen discontinuation should be accompanied by a close follow-up on the hormonal side to control for progestogen arrest-related side effects and on the neurosurgical side to manage the meningioma. Meningioma-related symptoms regularly regress as the tumour shrinks after progestogen withdrawal, which allows proposing a conservative management in most cases.^41^ A close clinical and imaging follow-up is generally warranted in this setting, but our data do not justify systematic MRI screening of all women receiving progestogens in the absence of a known meningioma; decisions about population-level screening programmes should remain the remit of guideline panels and health authorities, integrating local epidemiology, resources, and competing priorities. For hormonal alternatives, menopausal hormonal treatments and contraceptives should prioritise micronised progesterone with the knowledge that desogestrel is associated with a slight increased risk of meningiomas. In transgender women, discontinuing high dose progestogens and switching to alternatives like spironolactone is recommended.

Among the limitation, between-study heterogeneity was substantial for CPA and MPA, limiting precision; we therefore used random-effects models and performed leave-one-out checks. For DMPA, the high I^2^ and wide prediction interval indicate that the magnitude of excess risk varies across populations and exposure patterns. Evidence is also limited by restriction to English/French publications, the observational design with residual confounding, some inconsistency (e.g., uncertain post-withdrawal growth), and sparse long-term follow-up. Duration and cumulative dose of exposure were not always reported in a comparable way, which may attenuate or blur dose–response patterns and limits our ability to fully disentangle short-term from long-term effects. Randomised trials assigning women to long-term progestogen exposure would be ethically and logistically unrealistic; in practice, the most informative designs are large, well-curated cohorts or registry-based studies with detailed time-updated drug histories, rigorous outcome validation, and, ideally, target trial emulation frameworks for specific clinical questions. Unmeasured confounding remains a concern, especially for modest associations: for high-dose CPA and some DMPA exposures, an unmeasured confounder would need to be extremely strong and strongly imbalanced to fully explain 10–12-fold risk increases, whereas for small excess risks (e.g., relative risks around 1.2–1.3 for some oral regimens), even moderate residual confounding could partly or completely account for the observed associations. In addition, the review protocol was not prospectively registered (e.g., in PROSPERO), which may increase the risk of reporting bias despite the use of prespecified methods. Accordingly, certainty is moderate for CPA and low for MPA (GRADE), and interpretation emphasises direction of effect and the range of plausible magnitudes rather than a single pooled value. In several registry analyses, progestogens were given within oestrogen-progestin combinations; thus some “progestogen” contrasts also include oestrogen, complicating causal attribution.

In patients treated by several macroprogestogens and even in one microprogestogen, there is a significantly increased risk of intracranial meningioma. Clear, fair, and appropriate explanations are required before prescribing high-dose or long-term macroprogestogens. In patients with known or suspected meningioma, or with prolonged high-dose exposure, close clinical and imaging follow-up is advisable, in line with national and international guidance, rather than systematic MRI screening of all individuals using progestogens. Further research, including prospective studies, is necessary to assess if progestogen intake led to the growth of preexistent meningioma or to the apparition of meningioma.

## Contributors

BH, AR, and JP had access to and verified all the underlying study data.

BH, AR, and JP designed the study and developed the methodology.

BH and AR did the literature search and data curation.

BH and IDG performed the statistical analyses, with input from AR and JP.

All authors contributed to data interpretation, critically revised the manuscript for important intellectual content, and approved the final version for submission.

BH and JP had final responsibility for the decision to submit the manuscript for publication.

## Data sharing statement

De-identified extracted data and analytic code will be available with publication to qualified researchers upon reasonable request to the corresponding author for replication or secondary analyses, subject to approval of a proposal and signature of a data access agreement.

## Editor note

The Lancet Group takes a neutral position with respect to territorial claims in published maps and institutional affiliations.

## Declaration of interests

All authors declare no competing interests.

## Appendix A Supplementary data

Supplementary Data

Summary_French
