# Supplementary material for: Progestogen use and the risk of intracranial meningioma: a systematic review and meta-analysis
Source: eClinicalMedicine. 2026 Feb 12;92:103791. doi: 10.1016/j.eclinm.2026.103791 (PMC12925135; doi:10.1016/j.eclinm.2026.103791)
Supplement: Supplementary Data [file mmc1.pdf]

**Supplement data 1.** World map of the authorized countries for progestogens associated with meningiomas.

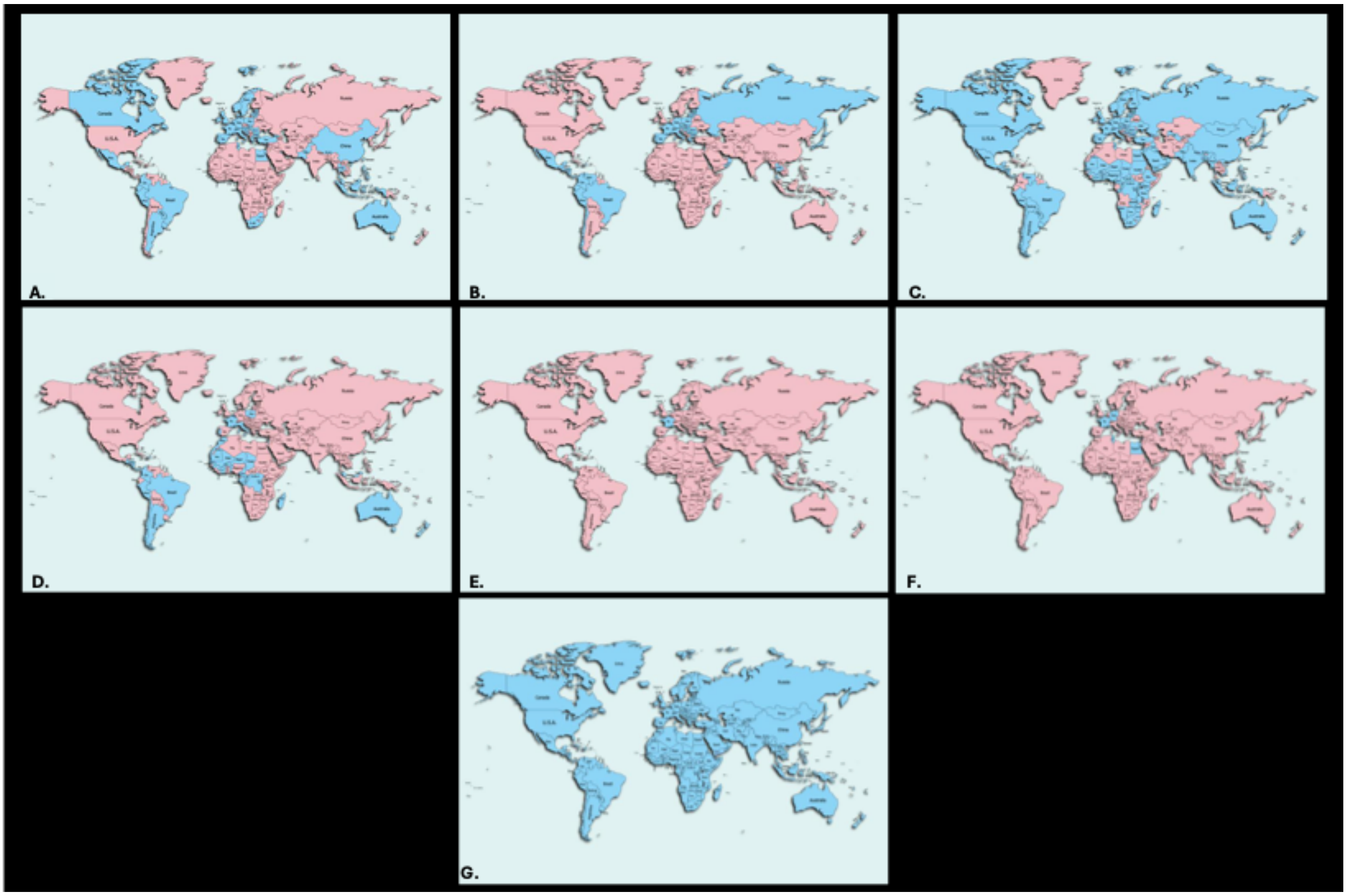


Each panel depicts one progestogen. Countries in blue indicate that the drug has marketing authorization, and countries in red indicate that it does not. Authorization status (which does not necessarily reflect current marketing) was obtained from national regulatory agency databases and drugs.com (last accessed December 2024); further details are provided in Supplementary data 11.

A. Cyproterone acetate. B. Chlormadinone acetate. C. Medroxyprogesterone acetate. D. Nomegestrol acetate. E. Promegestone. F. Medrogestone. G. Desogestrel.

## **Supplement data 2.** Search strategy

| **Search strategy on the US National Library of Medicine (PubMed/MEDLINE)** |
|---|
| *(((("Pregnanes"[Mesh])) OR "Hormones, Hormone Substitutes, and Hormone Antagonists"[Mesh]) AND "Meningioma"[Mesh])* |

| **Search strategy on Embase (OVID)** |
|---|
| *(pregnanes.mp. OR exp hormone/ OR exp hormone_antagonist/) AND exp meningioma/* |

| **Search strategy on Cochrane Library** |
|---|
| *(Pregnanes OR hormone* OR "hormone antagonists") AND meningioma* |

| **Search strategy on EPI-PHARE Scientific Interest Group of the French National Agency for Medicines and Health Products Safety database** |
|---|
| *Using the keywords "Meningiomas", "Méningiomes", "Pregnanes", and "Progesterone"* |

**Supplement data 3.** Steroidogenesis

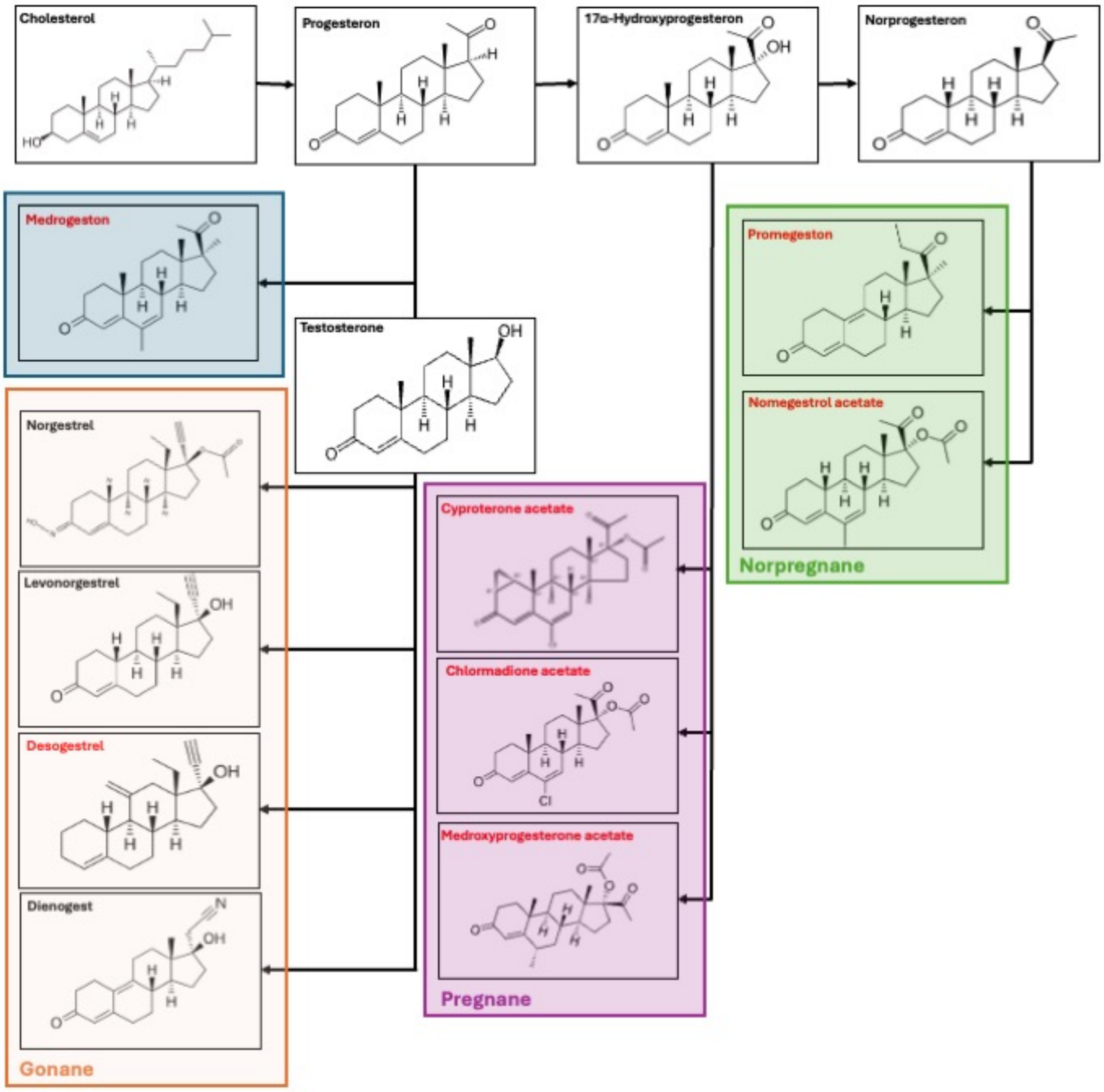


Cholesterol is the molecule that initiates the steroidogenesis cycle, leading, through several intermediates, to the production of progesterone. From there, Medrogestone (in blue) is obtained after transformation via 17-methylprogesterone as an intermediate. Progesterone is also one of the possible pathways for the production of testosterone, from which Gonanes (in orange) are derived. These include multiple microprogestins used in medical practice, such as Norgestrel, Levonorgestrel, Desogestrel, and Dienogest. Additionally, progesterone can give rise to 17α-Hydroxyprogesterone, leading to the Pregnanes (in purple), which include Cyproterone acetate, Chlormadinone acetate, and Medroxyprogesterone acetate. Norprogesterone is also derived from progesterone and enables the synthesis of Norpregnanes (in green), including Promegestone and Nomegestrol acetate.

**Supplement data 4.** Classification of Synthetic Progestogens

Synthetic progestogen modes of action depend on their class, which is determined by the hormone from which they are derived (Table 2 and Supplement data 6). In this review, we use the term "macroprogestogens" to describe high-dose systemic formulations (typically ≥5-10 mg/day orally or depot injections) most often prescribed for non-contraceptive indications (e.g., androgen-dependent conditions, endometriosis, oncology), and "microprogestogens" to describe low-dose progestins primarily used for contraception (e.g., norsteroid-derived progestins at standard contraceptive doses), in line with previous pharmacological classifications[1,2]. Progestogens from the pregnane, norpregnane, and medrogestone group are used at high doses and are termed "macroprogestogens" in this framework. Progestogens derived from norsteroids are used at lower doses and are termed "microprogestogens"[1,2].

*Pregnanes (derived from 17-hydroxyprogesterone)*

CPA acts as a peripheral anti-androgen and anti-gonadotroph. In men, CPA is indicated to treat prostate cancer, for gender transition therapy and for managing paraphilias. In women, CPA is indicated for severe hirsutism, endometriosis, severe acne, and was used as a contraceptive. Depending on the indication and country, doses range from 10-50 mg daily for systemic treatment, with continuous (28 days) or sequential (21 days on, seven days off) regimens; a 100 mg formulation has also been used historically in some settings. It also exists in a microdose form (2 mg) combined with ethinylestradiol for contraception[3].

Chlormadinone Acetate (CMA) primary action is luteomimetic, has anti-oestrogenic activity, moderate anti-gonadotropic effects, and lacks androgenic effects. In women, CMA is indicated for menstrual irregularities, premenstrual syndrome, dysmenorrhoea, mastodynia, artificial cycle regulation, contraception, functional bleeding, bleeding related to uterine fibroids and for the treatment of endometriosis[1]. Doses range 5-10 mg daily, with continuous or sequential treatment regimens, either as monotherapy or in combination with an oestrogen[1]. A 2 mg dose combined with ethinylestradiol is used in combined oral contraceptives in several country as Italy[4]; higher-dose (5-10 mg) tablets have been progressively restricted or withdrawn in some European countries since 2024[5].

Medroxyprogesterone acetate (MPA) acts as a potent progestogenic. This long-acting synthetic progestogen exerts antineoplastic effects in advanced endometrial and renal carcinoma. At oncological doses, corticotropic activity may occur. MPA is marketed both as an oral tablet (2.5-10 mg) for abnormal uterine bleeding (AUB) and various gynaecologic uses, and as long-acting contraceptive depot formulations (150 mg intramuscular and 104 mg/0.65

mL subcutaneous injections, collectively termed DMPA), which are widely used in family planning programmes worldwide (e.g., Southeast Asia, Indonesia, and the USA). In many settings DMPA represents a major, and sometimes first-line, long-acting reversible contraceptive method rather than a purely second-line option[1,2,6].

*Norpregnanes (derived from 17-alpha-norprogesterone or 19-norprogesterone)*

Nomegestrol Acetate (NOMAC) has no androgenic or oestrogenic activity and has approximately 2.5-fold higher affinity for progesterone receptors than the natural hormone. NOMAC is indicated for AUB, including that related to uterine fibroids, menstrual cycle disorders, and hormone replacement therapy for menopause. Doses range 2-5 mg daily, with continuous or sequential treatment regimens. In contraception, 2.5 mg NOMAC is combined with estradiol in a monophasic combined oral contraceptive pill[7].

Promegestone has a 5 times more high affinity for progesterone receptors than the natural hormone. It exhibits potent progestational activity without androgenic effects at low doses. Promegestone is indicated in managing menstrual irregularities, endometriosis, premenstrual syndrome, contraception and as part of hormone replacement therapy. Doses range 0.5-1 mg daily, with continuous or sequential treatment regimens[1,2,8]. Promegestone was primarily marketed in France and has been withdrawn from the market since 2020; its current use is therefore historical in most jurisdictions.

*Medrogestone (derived from 17-methylprogesterone)*

Medrogestone has no androgenic effects, has anti-oestrogenic properties, and may exhibit moderate anti-gonadotropic effects depending on the dose. Commercialised mostly in 5 mg tablets, it is primarily used in hormone replacement therapy for menopause, as well as in the treatment of endometriosis, dysmenorrhoea, and as a contraception. In several countries, 5 mg tablets have been discontinued, and current availability is mainly limited to low-dose preparations in combination with oestrogens for menopausal hormone therapy[1,2,9].

*Gonane (derived from testosterone)*

Norgestrel and its active form levonorgestrel exhibit progestogenic and anti-gonadotropic activity at sufficient doses[10]. Desogestrel has stronger anti-gonadotropic activity than norgestrel and lower androgenic activity. It is primarily used in contraception. Desogestrel 75 µg once daily is used for progestogen-only contraception, with strong antigonadotropic activity and low androgenicity. Levonorgestrel is also widely used in intrauterine systems

that provide long-acting local and systemic progestin exposure. Dienogest 2 mg once daily (continuous) is indicated for endometriosis and contraception[1,2,11].

## References


1 Schindler AE, Campagnoli C, Druckmann R, *et al.* Classification and pharmacology of progestins. *Maturitas* 2003; 46: 7.

2 Edwards M, Can AS. Progestins. StatPearls. 2024; published online Jan 10. http://www.ncbi.nlm.nih.gov/books/NBK563211/ (accessed Nov 1, 2025).

3 Cyprotérone : substance active à effet thérapeutique - VIDAL. https://www.vidal.fr/medicaments/substances/cyproterone-6753.html (accessed Jan 25, 2025).

4 Grandi G, Marani G, Facchinetti F, Bahamondes L. We don´t have elements to scare women who use oral contraceptives based on nomegestrol or chlormadinone about the risk of meningioma. Let's be careful and honest! *The European Journal of Contraception & Reproductive Health Care* 2022. DOI:10.1080/13625187.2022.2114792.

5 Chlormadinone : substance active à effet thérapeutique - VIDAL. https://www.vidal.fr/medicaments/substances/chlormadinone-15547.html# (accessed Jan 25, 2025).

6 Médroxyprogestérone : substance active à effet thérapeutique - VIDAL. https://www.vidal.fr/medicaments/substances/medroxyprogesterone-6825.html (accessed Jan 25, 2025).

7 Nomégestrol : substance active à effet thérapeutique - VIDAL. https://www.vidal.fr/medicaments/substances/nomegestrol-6839.html# (accessed Jan 25, 2025).

8 Promégestone : substance active à effet thérapeutique - VIDAL. https://www.vidal.fr/medicaments/substances/promegestone-2932.html (accessed Jan 25, 2025).

9 Médrogestone : substance active à effet thérapeutique - VIDAL. https://www.vidal.fr/medicaments/substances/medrogestone-2251.html# (accessed Jan 25, 2025).

10 Norgestrel : substance active à effet thérapeutique - VIDAL. https://www.vidal.fr/medicaments/substances/norgestrel-6841.html (accessed Jan 25, 2025).

11 Diénogest : substance active à effet thérapeutique - VIDAL. https://www.vidal.fr/medicaments/substances/dienogest-21941.html (accessed Jan 25, 2025).

**Supplement data 5.** Leave-one-out analyses for included studies in meta-analysis

| Model | N | Risk (95% CI) | $I^2$ | $Tau^2$ | PI 95% |
|---|---|---|---|---|---|
| **CPA** | | | | | |
| All studies | 5 | 12.36 (7.47-20.45) | 73.8 | 0.2044 | 4.46-34.26 |
| Minus Gil et al. (2011) | 4 | 12.35 (6.73-22.64) | 81.2 | 0.2694 | 3.78-40.35 |
| Minus Cea-Soriano et al. (2012) | 4 | 13.11 (7.69-22.36) | 78.7 | 0.2129 | 4.59-37.47 |
| Minus Weill et al. (2021) | 4 | 18.03 (15.78-20.61) | 0.0 | 0.000 | 15.78-20.61 |
| Minus Mikkelsen et al. (2022) | 4 | 10.81 (5.88-19.86) | 75.5 | 0.2450 | 3.44-33.97 |
| Minus Hoisnard et al. (2022) | 4 | 10.32 (5.65-18.86) | 55.9 | 0.1981 | 3.57-29.80 |
| **DMPA** | | | | | |
| All studies | 6 | 2.68 (1.72-4.19) | 92.7 | 0.2504 | 0.91-7.88 |
| Minus Roland et al. (2024) | 5 | 2.45 (1.54-3.90) | 93.7 | 0.2419 | 0.84-7.14 |
| Minus Griffin et al. (2024) | 5 | 3.03 (1.85-4.94) | 85.3 | 0.2399 | 1.03-8.89 |
| Minus Griffin et al. (2025) | 5 | 2.83 (1.70-4.71) | 94.6 | 0.2905 | 0.88-9.15 |
| Minus Xiao et al. (2025) | 5 | 2.75 (1.59-4.78) | 94.2 | 0.3257 | 0.79-9.58 |
| Minus Tettamanti et al. (2025) | 5 | 2.08 (1.58-2.74) | 59.3 | 0.0491 | 1.25-3.48 |
| Minus Reynolds et al. (2025) | 5 | 2.91 (1.74-4.86) | 94.1 | 0.2811 | 0.91-9.28 |

**Supplement data 6.** Code on R for meta-analysis

```
library(readxl)
library(dplyr)
library(metafor)

path_cpa <- "~/Library/Mobile Documents/com~apple~CloudDocs/Articles/Meningiome et Hormones/Meta analyse/RIsk/CPA_v2.xlsx"

dat_cpa <- read_excel(path_cpa)

print(dat_cpa)

dat_cpa <- dat_cpa |>
  mutate(
    log_effect = log(Effect),
    se         = (log(Upper_CI) - log(Lower_CI)) / (2 * 1.96),
    vi         = se^2
  )

print(dat_cpa)

m_cpa <- rma(
  yi     = log_effect,
  vi     = vi,
  data   = dat_cpa,
  method = "REML",
  slab   = Study
)

summary(m_cpa)

pred_cpa <- predict(m_cpa, transf = exp)

cat("\nEffet poolé (échelle ratio) :\n")
cat("Pooled =", round(pred_cpa$pred, 2),
    " [", round(pred_cpa$ci.lb, 2), ";", round(pred_cpa$ci.ub, 2), "]\n")

cat("\nHétérogénéité :\n")
cat("Tau^2 =", round(m_cpa$tau2, 3), "\n")
cat("I^2   =", round(m_cpa$I2, 1), "%\n")

forest(
  m_cpa,
  atransf = exp,
  refline = 1,
  xlab = "Risque relatif (OR/HR/RR) - CPA forte dose",
  at = log(c(1, 2, 5, 10, 20))
)

w_rand <- weights(m_cpa)

m_cpa_fe <- rma(
  yi     = log_effect,
  vi     = vi,
  data   = dat_cpa,
  method = "FE",
  slab   = Study
)

w_fixed <- weights(m_cpa_fe)

weights_df <- data.frame(
  Study             = dat_cpa$Study,
  weight_random     = round(w_rand, 2),
  weight_random_pct = round(w_rand / sum(w_rand) * 100, 1),
  weight_fixed      = round(w_fixed, 2),
  weight_fixed_pct  = round(w_fixed / sum(w_fixed) * 100, 1)
)

print(weights_df)
```

**Supplement data 7.** Sensitivity analysis of RR and HR conversion

| Model | Original risk metric | Initial risk (95%CI) | Converted | Baseline risk $p_0$ (per person-year) | Converted in OR (95%CI) |
|---|---|---|---|---|---|
| **CPA** | | | | | |
| Gil et al. (2011) | RR | 11.4 (4.3-30.8) | Yes | 0.000066 | 11.41 (4.30–30.86) |
| Cea-Soriano et al. (2012) | OR | 6.30 (1.37-28.94) | No | / | / |
| Weill et al. (2021) | HR | 6.6 (4.0-11.1) | Yes | 0.000045 | 6.60 (4.00–11.11) |
| Mikkelsen et al. (2022) | HR | 19.2 (10.3-35.8) | Yes | 0.000093 | 19.23 (10.31–35.92) |
| Hoisnard et al. (2022) | OR | 18.3 (16.0-21.1) | No | / | / |
| **DMPA** | | | | | |
| Griffin et al. (2024) | OR | 1.68 (1.50-1.87) | No | / | / |
| Roland et al. (2024) | OR | 5.55 (2.27-13.56) | No | / | / |
| Tettamanti et al. (2025) | OR | 5.49 (4.51-6.67) | No | / | / |
| Reynolds et al. (2025) | OR | 1.81 (1.14-2.89) | No | / | / |
| Griffin et al. (2025) | OR | 1.91 (0.99-4.50) | No | / | / |
| Xiao et al. (2025) | RR | 2.43 (1.77-3.33) | Yes | 0.00062 | 2.43 (1.77-3.34) |

**Supplement data 8.** Newcastle-Ottawa Scale (NOS) scoring for included studies in meta-analysis.

| Study | Design | Selection | Comparability | Exposure/Outcome | Total |
|---|---|---|---|---|---|
| *Gil et al. (2011)* | Cohort | 4 | 2 | 3 | 9 |
| *Griffin et al. (2024)* | Case-control | 4 | 2 | 3 | 9 |
| *Hoisnard et al. (2022)* | Case-control | 4 | 2 | 3 | 9 |
| *Mikkelsen et al. (2022)* | Cohort | 4 | 2 | 3 | 9 |
| *Nguyen et al. (2024)* | Cohort | 4 | 2 | 3 | 9 |
| *Roland et al. (2024)* | Case-control | 4 | 2 | 3 | 9 |
| *Roland et al. (2024)* | Case-control | 4 | 2 | 3 | 9 |
| *Weill et al. (2021)* | Cohort | 4 | 2 | 3 | 9 |
| *Cea-Soriano et al. (2012)* | Case-control | 4 | 2 | 3 | 9 |
| *Epi-Phare et al. (2024)* | Case-control | 4 | 2 | 3 | 9 |
| *Xiao et al. (2025)* | Cohort | 4 | 2 | 3 | 9 |
| *Reynold et al. (2025)* | Case-control | 4 | 2 | 3 | 9 |
| *Tettamanti et al. (2025)* | Case-control | 4 | 2 | 3 | 9 |
| *Griffin et al. (2025)* | Case-control | 4 | 2 | 3 | 9 |

**Supplement data 9.** Grading of Recommendations Assessment, Development and Evaluation (GRADE) scoring for included studies in meta-analysis.

| Exposure | Comparator | Relative effect | Studies (n) | Heterogeneity ($I^2$) | Grading | Certainty (GRADE) |
|---|---|---|---|---|---|---|
| Cyproterone acetate (CPA) | No exposure | 12.36 (7.47–20.45) | 5 | 73.8% | Starting level:<br>1) Observational evidence: LOW<br>2) Very large effect: +2<br>3) Dose-response: +1<br>4) Residual confounding*: −1 | MODERATE |
| Depot medroxyprogesterone acetate (DMPA) | No exposure | 2.68 (1.72-4.19) | 6 | 92.7% | Starting level:<br>1) Observational evidence: LOW<br>2) Large effect: +1<br>3) Dose-response: +1<br>4) Residual confounding[$]: −1<br>5) Inconsistency[£]: -1 | LOW |
| Chlormadinone acetate (CMA) | No exposure | 3.10 (2.40-4.00) | 3 | NA (not pooled) | Starting level:<br>1) Observational evidence: LOW<br>2) Large effect: +1<br>3) Dose-response: +1<br>4) Residual confounding: −1<br>5) clinical/methodological heterogeneity**: -1 | LOW |
| Nomegestrol acetate (NOMAC) | No exposure | 2.90 (2.40-3.70) | 3 | NA (not pooled) | Starting level:<br>1) Observational evidence: LOW<br>2) Large effect: +1<br>3) Dose-response: +1<br>4) Residual confounding: −1<br>5) clinical/methodological heterogeneity **: -1 | LOW |
| Medrogestone | No exposure | 3.49 (2.38–5.10) | 1 | NA (not pooled) | Starting level:<br>1) Observational evidence: LOW<br>2) Large effect: +1<br>3) Dose-response: +1<br>4) Residual confounding: −1 | LOW |
| Promegestone | No exposure | 2.39 (1.85–3.09) | 1 | NA (not pooled) | Starting level:<br>1) Observational evidence: LOW<br>2) Large effect: +1<br>3) Dose-response: +1<br>4) Residual confounding: −1 | LOW |
| Desogestrel | No exposure | 1.25 (1.10–1.42) | 2 | NA (not pooled) | Starting level:<br>1) Observational evidence: LOW<br>2) Dose-response: +1<br>3) Residual confounding: −1<br>4) clinical/methodological heterogeneity**: -1 | VERY LOW |

**Downgraded one level due to unmeasured reproductive factors (parity, menopausal status), adiposity, concomitant hormonal exposures, and potential indication/channeling and detection biases not fully captured in administrative data; although a surgery/radiotherapy endpoint and dose-response mitigate detection bias and support causality, residual confounding remains plausible*
*[$]Downgraded one level for unmeasured reproductive/clinical factors, SES and race/ethnicity correlates of DMPA use, and exposure misclassification (injection timing/adherence).*
*[£]Downgraded one level due to between-study differences in magnitude (US claims vs French data with very few exposed cases) and limited number of studies, leading to uncertainty about the true effect size.*
*** Downgraded one level for clinical/methodological heterogeneity due to between-study differences in study design and effect estimates, including variations in data sources (registry vs claims), exposure definitions (dose/duration ascertainment), outcome ascertainment (diagnosis vs surgery/radiotherapy-confirmed meningioma), comparator definitions, and adjustment strategies, limiting confidence that a single underlying effect applies across studies and for small effect sizes (eg, desogestrel), residual confounding may plausibly explain the association.*

*NA: CPA inconsistency not downgraded. Despite high $I^2$, all estimates point in the same direction with very large magnitude; heterogeneity is plausibly explained by differences in endpoint definition (diagnosis vs surgery/radiotherapy), dose ascertainment, and populations; the 95% prediction interval remains well above 1.*

**Supplement data 10** Article list for systematic review

| Author | Title |
|---|---|
| *Shimizu et al. (2008)* | Spontaneous regression of an asymptomatic meningioma associated with discontinuation of progesterone agonist administration. |
| *Champeaux-Depond et al. (2021)* | Cyproterone acetate and meningioma: a nationwide-wide population based study. |
| *Cea-Soriano et al. (2012)* | Hormonal therapies and meningioma: is there a link? |
| *Roland et al. (2024)* | Use of progestogens and the risk of intracranial meningioma: national case-control study. |
| *Hoisnard et al. (2022)* | Risk of intracranial meningioma with three potent progestogens: A population-based case-control study. |
| *Goncalves et al. (2010)* | Abrupt regression of a meningioma after discontinuation of cyproterone treatment |
| *Bergoglio et al. (2012)* | Symptomatic meningioma induced by cross-sex hormone treatment in a male-to-female transsexual |
| *Borghei-Razavi et al. (2014)* | Meningioma: The Unusual Growth in a Transsexual Patient after Estrogen-Progesterone Therapy |
| *Botella et al. (2015)* | Méningiomes intracrâniens et utilisation prolongée d'acétate de cyprotérone à dose conventionnelle chez la femme : à propos de deux cas de régression tumorale après arrêt du traitement |
| *Abou-al-shaar et al. (2023)* | Skull Base Meningiomas as Part of a Novel Meningioma Syndrome Associated with Chronic Depot Medroxyprogesterone Acetate Use |
| *Bernat et al. (2018)* | Regression of Giant Olfactory Groove Meningioma and Complete Visual Acuity Recovery after Discontinuation of Cyproterone Acetate |
| *Alderman et al. (2016)* | Probable Drug-Related Meningioma Detected During the Course of Medication Review Services |
| *Roland et al. (2024)* | Prolonged use of chlormadinone acetate and risk of intracranial meningioma: A population-based cohort study. |
| *Nguyen et al. (2024)* | Prolonged use of nomegestrol acetate and risk of intracranial meningioma: a population-based cohort study |
| *AlDoheyan et al. (2024)* | Regression of multiple intracranial meningiomas after cessation of long-term synthetic progesterone (megestrol) medication: case report and autopsy. |
| *Gazzeri et al. (2007)* | Growth of a meningioma in a transsexual patient after estrogen-progestin therapy. |
| *Cebula et al. (2010)* | Regression of meningiomas after discontinuation of cyproterone acetate in a transsexual patient. |
| *Rapport Epi-Phare (2024)* | Contraception orale progestative et risque de méningiome intracrânien Une étude cas-témoins à partir des données du système national des données de santé |
| *De Germay et al. (2021)* | Meningiomas after cyproterone acetate exposure: Case reports in twin sisters. |
| *Murata et al. (2003)* | Meningioma in a woman receiving hormone therapy. |
| *Benson et al. (2015)* | Menopausal hormone therapy and central nervous system tumor risk: large UK prospective study and meta-analysis. |
| *Pourhadi et al. (2023)* | Menopausal hormone therapy and central nervous system tumors: Danish nested case-control study. |
| *Ter et al. (2016)* | Meningiomas in three male-to-female transgender subjects using oestrogens/progestogens and review of the literature. |
| *Gruber et al. (2004)* | Multiple meningiomas arising during long-term therapy with the progesterone agonist megestrol acetate. Case report. |
| *Michaud et al. (2010)* | Reproductive factors and exogenous hormone use in relation to risk of glioma and meningioma in a large European cohort study |
| *Wigertz et al. (2006)* | Risk of brain tumors associated with exposure to exogenous female sex hormones. |
| *Champagne et al. (2019)* | Combined hormonal influence of cyproterone acetate and nomegestrol acetate on meningioma: a case report. |
| *Jhawar et al. (2003)* | Sex steroid hormone exposures and risk for meningioma. |
| *Griffin et al. (2024)* | The Association between Medroxyprogesterone Acetate Exposure and Meningioma |
| *Roux et al. (2020)* | Symptomatic progestin-associated atypical grade II meningioma. A first case report. |
| *QI et al. (2013)* | Reproductive and exogenous hormone factors in relation to risk of meningioma in women: a meta-analysis. |
| *Korhonen et al. (2012)* | A nationwide cohort study on the incidence of meningioma in women using postmenopausal hormone therapy in Finland. |
| *Millward et al. (2021)* | Gender-affirming hormone therapy associated with multiple meningiomas and atypical histology in a transgender woman. |
| *Batchinsky-Parrou et al. (2022)* | First case of cyproterone acetate induced multiple meningiomas in identical female twins: A case report. |
| *Grandi et al. (2022)* | We don´t have elements to scare women who use oral contraceptives based on nomegestrol or chlormadinone about the risk of meningioma. Let's be careful and honest! |
| *Korhonen et al. (2010)* | Exogenous sex hormone use and risk of meningioma: a population-based case-control study in Finland. |
| *Shahar et al. (2011)* | Hormonal effect on meningioma growth. |
| *Custer et al. (2006)* | Hormonal exposures and the risk of intracranial meningioma in women: a population-based case-control study |
| *Mancini et al. (2018)* | Presentation of a meningioma in a transwoman after nine years of cyproterone acetate and estradiol intake: case report and literature review. |
| *Graillon et al. (2021)* | Meningiomas in patients with long-term exposition to progestins: Characteristics and outcome. |
| *Comité technique de pharmacovigilance (2019)* | Comité technique de pharmacovigilance : Enquête concernant les méningiomes et l'acétate de cyprotérone |
| *Ahmed-Khalifa et al. (2023)* | Radiological evolution of progestogen-induced meningioma: A monocentric retrospective study. |
| *Devalckeneer et al. (2021)* | Preliminary report of patients with meningiomas exposed to Cyproterone Acetate, Nomegestrol Acetate and Chlormadinone Acetate - Monocentric ongoing study on progestin related meningiomas. |

| | |
|---|---|
| *Vadivelu et al. (2010)* | Regression of multiple intracranial meningiomas after cessation of long-term progesterone agonist therapy. |
| *Gruber et al. (2011)* | Intracranial meningiomas in patients with uterine sarcoma treated with long-term megestrol acetate therapy. |
| *Frassanito et al. (2012)* | Hormonal therapy for fertility and huge meningioma: a purely random association? |
| *Nota et al. (2018)* | The occurrence of benign brain tumours in transgender individuals during cross-sex hormone treatment. |
| *Samarut et al. (2021)* | Meningiomas and cyproterone acetate: a retrospective, monocentric cohort of 388 patients treated by surgery or radiotherapy for intracranial meningioma. |
| *Passeri et al. (2022)* | Atypical evolution of meningiomatosis after discontinuation of cyproterone acetate: clinical cases and histomolecular characterization. |
| *Harland et al. (2018)* | Progesterone-only contraception is associated with a shorter progression-free survival in premenopausal women with WHO Grade I meningioma. |
| *Bernat et al. (2015)* | Growth stabilization and regression of meningiomas after discontinuation of cyproterone acetate: a case series of 12 patients. |
| *Passeri et al. (2019)* | Spontaneous regression of meningiomas after interruption of nomegestrol acetate: a series of three patients. |
| *Malaize et al. (2021)* | Evolution of the neurosurgical management of progestin-associated meningiomas: a 23-year single-center experience. |
| *Apra et al. (2020)* | Female gender and exogenous progesterone exposition as risk factors for spheno-orbital meningiomas. |
| *Florea et al. (2023)* | Opposed evolution of the osseous and soft parts of progestin-associated osteomeningioma after progestin intake discontinuation. |
| *AbiJaoude et al. (2021)* | Sustained growth of intraosseous hormone-associated meningiomas after cessation of progestin therapy. |
| *Voormolen et al. (2021)* | Intracranial Meningiomas Decrease in Volume on Magnetic Resonance Imaging After Discontinuing Progestin. |
| *Peyre et al. (2018)* | Progestin-associated shift of meningioma mutational landscape |
| *Raport Epi-Phare et al. (2022)* | Évaluation de l'impact des mesures de réduction du risque de méningiomes intracrâniens lié à l'utilisation de fortes doses d'acétate de cyprotérone en France |
| *Froelich et al. (2008)* | Does cyproterone acetate promote multiple meningiomas ? |
| *Samoyeau et al. (2022)* | Meningioma in patients exposed to progestin drugs: results from a real-life screening program. |
| *Gil et al. (2011)* | Risk of meningioma among users of high doses of cyproterone acetate as compared with the general population: evidence from a population-based cohort study. |
| *Weill et al. (2021)* | Use of high dose cyproterone acetate and risk of intracranial meningioma in women: cohort study. |
| *Mikkelsen et al. (2022)* | Cyproterone acetate and risk of meningioma: a nationwide cohort study. |
| *Sweeney et al. (2024)* | Physician Awareness of the Safe Use of Cyproterone Acetate in Europe: A Survey on the Effectiveness of Additional Risk Minimization Measures. |
| *Lee et al. (2022)* | A systematic review and meta-analysis of the association between cyproterone acetate and intracranial meningiomas. |
| *ANSM et al. (2024)* | De nouvelles données sur le risque de méningiome associé à la prise de progestatifs en contraception orale |
| *Davis et al. (2011)* | Progesterone and meningiomas. |
| *Girardelli et al. (2022)* | Meningiomas in Gynecology and Reproduction: an Updated Overview for Clinical Practice. |
| *Roux et al. (2023)* | Chlormadinone acetate-associated grade 3 anaplastic meningioma. |
| *Vercellini et al. (2023)* | Nomegestrol acetate for symptomatic endometriosis and meningioma risk: understandable statistics for decision-making. |
| *Krause et al. (2024)* | Progestin-associated meningiomatosis with unusual schwannoma-like morphology. |
| *Hage et al. (2022)* | Estrogen and Progesterone Therapy and Meningiomas. |
| *Roland et al. (2025)* | Oral contraceptives with progestogens desogestrel or levonorgestrel and risk of intracranial meningioma: national case-control study |
| *Xiao et al. (2025)* | *Depot Medroxyprogesterone Acetate and Risk of Meningioma in the US* |
| *Reynolds et al. (2025)* | *The Association Between Medroxyprogesterone Acetate Exposure and Cerebral Meningioma Among a Medicaid Population* |
| *Tettamanti et al. (2025)* | *Hormonal contraceptives and the risk of meningioma: a Swedish register-based case-control study* |
| *Griffin et al. (2025)* | *A Matched Case-Control Study Examining the Association Between Exposure to Depot Medroxyprogesterone Acetate and Cerebral Meningioma Using an Active Comparator* |

**Supplement data 11.** Article list for meta-analysis

| Author | Title |
|---|---|
| *Gil et al. (2011)* | Risk of meningioma among users of high doses of cyproterone acetate as compared with the general population: evidence from a population-based cohort study. |
| *Griffin et al. (2024)* | The Association between Medroxyprogesterone Acetate Exposure and Meningioma |
| *Hoisnard et al. (2022)* | *Risk of intracranial meningioma with three potent progestogens: A population-based case-control study.* |
| *Mikkelsen et al. (2022)* | Cyproterone acetate and risk of meningioma: a nationwide cohort study. |
| *Cea-Soriano et al. (2012)* | Hormonal therapies and meningioma: is there a link? |
| *Roland et al. (2024) Epi-phare* | Prolonged use of chlormadinone acetate and risk of intracranial meningioma: A population-based cohort study. |
| *Nguyen et al. (2024) Epi-phare* | Prolonged use of nomegestrol acetate and risk of intracranial meningioma: a population-based cohort study |
| *Weill et al. (2021) Epi-phare* | Use of high dose cyproterone acetate and risk of intracranial meningioma in women: cohort study. |
| *Roland et al. (2024) Epi-phare* | Use of progestogens and the risk of intracranial meningioma: national case-control study. |
| *Epi-Phare et al. (2024)* | Contraception orale progestative et risque de méningiome intracrânien Une étude cas-témoins à partir des données du système national des données de santé (desog) |
| *Xiao et al. (2025)* | *Depot Medroxyprogesterone Acetate and Risk of Meningioma in the US* |
| *Reynolds et al. (2025)* | *The Association Between Medroxyprogesterone Acetate Exposure and Cerebral Meningioma Among a Medicaid Population* |
| *Tettamanti et al. (2025)* | *Hormonal contraceptives and the risk of meningioma: a Swedish register-based case-control study* |
| *Griffin et al. (2025)* | *A Matched Case-Control Study Examining the Association Between Exposure to Depot Medroxyprogesterone Acetate and Cerebral Meningioma Using an Active Comparator* |

**Supplement data 12.** Full-text studies excluded, with reasons

| Author | Title | Reason |
|---|---|---|
| *Deipolyi (2010)* | *Development of a symptomatic intracranial meningioma in a male-to-female transsexual after initiation of hormone therapy.* | Hormonal exposure insufficiently specified (type/dose/duration and progestogen component not reported), therefore not extractable. |
| *Cowppli-Bony (2011)* | *Brain tumors and hormonal factors: review of the epidemiological literature.* | No primary, extractable data on meningioma outcomes with eligible systemic progestogens. |
| *Bernard-Weil et al (1963)* | *Preoperative hormonal treatment in cases of cerebral tumor* | Non-eligible exposure (preoperative “hormonal treatment” not corresponding to the systemic progestogens of interest / pregnane derivatives) and no extractable outcome for this review. |
| *Tsutsui (2016)* | *Acute Progression of Recurrent Meningioma during Luteinizing Hormone-Releasing Hormone Agonist Treatment for Prostate Cancer.* | Exposure was a GnRH/LHRH agonist (non-eligible exposure). |
| *Davis (1990)* | *Meningiomas and sex hormones.* | No primary extractable data with eligible exposures. |
| *Grunberg (1994)* | *Role of antiprogestational therapy for meningiomas.* | No primary extractable data with eligible exposures. |
| *Lee (2003)* | *Luteinizing hormone-releasing hormone agonists and meningioma: a treatment dilemma.* | Exposure was a GnRH/LHRH agonist (non-eligible exposure). |
| *Claus et al. (2007)* | *Exogenous hormone use and meningioma risk* | Discusses exogenous hormones in general without specifying eligible progestogens/exposures, no extractable data. |

# **Supplement data 13.** Risk of meningioma development

| Author | Title | Risk | N |
|---|---|---|---|
| ***Cyproterone Acetate*** | | | |
| *Gil et al. (2011)* | *Risk of meningioma among users of high doses of cyproterone acetate as compared with the general population: evidence from a population-based cohort study.* | RR: 11.4 (95%CI: 4.3-30.8) | 4 |
| *Cea-Soriano et al. (2012)* | *Hormonal therapies and meningioma: is there a link?* | OR: 6.30 (95%CI: 1.37-28.94) | 3 |
| *Weill et al. (2021)* | *Use of high dose cyproterone acetate and risk of intracranial meningioma in women: cohort study.* | HR: 6.6 (95%CI: 4.0-11.1) | 69 |
| *Mikkelsen et al. (2022)* | *Cyproterone acetate and risk of meningioma: a nationwide cohort study.* | HR: 19.2 (95%CI: 10.3-35.8) | 10 |
| *Hoisnard et al. (2022)* | *Risk of intracranial meningioma with three potent progestogens: A population-based case-control study.* | OR: 18.3 (95%CI: 16.0-21.1) | 961 |
| *Roland et al. (2024)* | *Use of progestogens and the risk of intracranial meningioma: national case-control study.* | OR: 19.2 (95%: 16.61-22.22) | 891 |
| ***Chlormadinone Acetate*** | | | |
| *Hoisnard et al. (2022)* | *Risk of intracranial meningioma with three potent progestogens: A population-based case-control study.* | OR: 4.7 (95%CI: 4.5-5.3) | 969 |
| *Roland et al. (2024)* | *Use of progestogens and the risk of intracranial meningioma: national case-control study.* | OR: 3.87 (95%CI: 3.48-4.30) | 628 |
| *Roland et al. (2024)* | *Prolonged use of chlormadinone acetate and risk of intracranial meningioma: A population-based cohort study.* | RR: 3.1 (95%CI: 2.4-4.0) | 164 |
| ***Medroxyprogesterone acetate*** | | | |
| *Roland et al. (2024)* | *Use of progestogens and the risk of intracranial meningioma: national case-control study.* | OR: 5.55 (95%CI : 2.27-13.56) | 9 |
| *Griffin et al. (2024)* | *The Association between Medroxyprogesterone Acetate Exposure and Meningioma* | OR: 1.68 (95%CI: 1.50-1.87) | 480 |
| *Xiao et al. (2025)* | *Depot Medroxyprogesterone Acetate and Risk of Meningioma in the US* | RR: 2.43 (95%CI: 1.77-3.33) | 131 |
| *Reynolds et al. (2025)* | *The Association Between Medroxyprogesterone Acetate Exposure and Cerebral Meningioma Among a Medicaid Population* | OR: 1.81 (95%CI: 1.14-2.89) | 29 |
| *Tettamanti et al (2025)* | *Hormonal contraceptives and the risk of meningioma: a Swedish register-based case-control study* | OR: 5.49 (95%CI: 4.51-6.67) | 186 |
| *Griffin et al. (2025)* | *A Matched Case-Control Study Examining the Association Between Exposure to Depot Medroxyprogesterone Acetate and Cerebral Meningioma Using an Active Comparator* | OR: 1.9 (95%CI: 0.99-4.5) | 7 |
| ***Nomegestrol Acetate*** | | | |
| *Hoisnard et al. (2022)* | *Risk of intracranial meningioma with three potent progestogens: A population-based case-control study.* | OR: 4.7 (95%CI: 4.3-5.1) | 969 |
| *Roland et al. (2024)* | *Use of progestogens and the risk of intracranial meningioma: national case-control study.* | OR : 4.93 (95%CI: 4.50-5.41) | 925 |
| *Nguyen et al. (2024)* | Prolonged use of nomegestrol acetate and risk of intracranial meningioma: a population-based cohort study | RR: 2.9 (95%CI: 2.4-3.7) | 171 |
| ***Promegestone*** | | | |
| *Roland et al. (2024)* | *Use of progestogens and the risk of intracranial meningioma: national case-control study.* | OR: 2.39 (95%CI : 1.85-3.09) | 83 |
| ***Medrogestone*** | | | |
| *Roland et al. (2024)* | *Use of progestogens and the risk of intracranial meningioma: national case-control study.* | OR: 3.49 (95%CI : 2.38-5.10) | 42 |
| ***Desogestrel*** | | | |
| Epi-phare (2024) | Contraception orale progestative et risque de méningiome intracrânien. Une étude cas-témoins à partir des données du système national des données de santé | OR: 1.25 (95%CI: 1.10-1.42) | 287 |
| *Roland et al. (2025)* | Oral contraceptives with progestogens desogestrel or levonorgestrel and risk of intracranial meningioma: national case-control study | OR: 1.32 (95% CI: 1.14-1.53). | 287 |

**Supplement data 14.** Countries where progestogens are authorized*

| Progestogens | Authorized country* |
|---|---|
| Cyproterone Acetate | Argentina, Austria, Australia, Belgium, Bosnia & Herzegowina, Brazil, Bulgaria, Canada, China, Colombia, Croatia (Hrvatska), Cyprus, Denmark, Ecuador, Egypt, Estonia, France, Georgia, Germany, Greece, Hong Kong, Hungary, Indonesia, Ireland, Italy, Kuwait, Latvia, Lebanon, Lithuania, Luxembourg, Macedonia, Malaysia, Malta, Mexico, Netherlands, Norway, Pakistan, Paraguay, Peru, Philippines, Poland, Portugal, Romania, Russian Federation, Serbia, Singapore, Slovakia, Slovenia, South Africa, Spain, Sweden, Switzerland, Taiwan, Thailand, Tunisia, Turkey, United Kingdom, Uruguay |
| Chlormadinone Acetate | Austria, Belgium, Brazil, Bulgaria, Chile, Colombia, Costa Rica, Croatia, Czech Republic, Dominican Republic, Ecuador, El Salvador, Estonia, France, Georgia, Germany, Greece, Guatemala, Honduras, Hungary, Israel, Italy, Japan, Latvia, Lithuania, Luxembourg, Mexico, Nicaragua, Oman, Panama, Peru, Poland, Portugal, Romania, Russia, Serbia, Slovakia, Spain, Switzerland, Thailand, Venezuela. |
| Medroxyprogesterone Acetate | Argentina, Australia, Bahamas, Bahrain, Bangladesh, Belize, Benin, Bolivia, Botswana, Brazil, Bulgaria, Burkina Faso, Burundi, Cameroon, Canada, Central African Republic, Chad, China, Congo, Costa Rica, Côte d'Ivoire, Curacao, Cyprus, Czech Republic, Denmark, Egypt, El Salvador, Estonia, Ethiopia, Finland, France, Georgia, Germany, Ghana, Guatemala, Honduras, Hungary, India, Indonesia, Iraq, Ireland, Israel, Italy, Jamaica, Japan, Jordan, Kenya, Kuwait, Latvia, Lebanon, Liberia, Lithuania, Luxembourg, Macao, Madagascar, Malawi, Malaysia, Mali, Malta, Mauritania, Mauritius, Mexico, Mongolia, Monaco, Myanmar, Namibia, Nepal, Netherlands, New Zealand, Nicaragua, Niger, Nigeria, Norway, Oman, Pakistan, Palestine, Panama, Paraguay, Peru, Philippines, Poland, Portugal, Qatar, Romania, Russia, Rwanda, Saudi Arabia, Senegal, Serbia, Singapore, Slovakia, Slovenia, South Africa, Spain, Sri Lanka, Sudan, Sweden, Switzerland, Taiwan, Tanzania, Togo, Trinidad and Tobago, Tunisia, Turkey, Uganda, Ukraine, United Arab Emirates, United Kingdom, USA, Uruguay, Venezuela, Vietnam, Yemen, Zambia, Zimbabwe. |
| Nomegestrol Acetate | Argentina, Australia, Austria, Belgium, Benin, Brazil, Burkina Faso, Cameroon, Chad, Chile, Colombia, Congo, Costa Rica, Croatia, Denmark, Dominican Republic, El Salvador, Finland, France, Gabon, Germany, Guatemala, Guinea, Honduras, Hungary, Ireland, Israel, Italy, Ivory Coast, Latvia, Lithuania, Luxembourg, Madagascar, Malaysia, Mali, Mauritania, Mauritius, Monaco, Morocco, the Netherlands, New Zealand, Nicaragua, Niger, Norway, Panama, Peru, Poland, Portugal, Russia, Senegal, Slovakia, Spain, Sweden, Switzerland, Togo, the United Kingdom. |
| Promegestone | France. |
| Medrogestone | Egypt, France, Germany, Tunisia. |
| Desogestrel | Afghanistan, Albania, Algeria, American Samoa, Andorra, Angola, Antigua and Barbuda, Argentina, Armenia, Australia, Austria, Azerbaijan, Bahrain, Bangladesh, Barbados, Belarus, Belgium, Belize, Benin, Bhutan, Bolivia, Bosnia and Herzegovina, Botswana, Brazil, Brunei, Bulgaria, Burkina Faso, Burundi, Cambodia, Cameroon, Canada, Cape Verde, Central African Republic, Chad, Chile, China, Colombia, Comoros, Costa Rica, Croatia, Cuba, Cyprus, Czech Republic, Democratic Republic of the Congo, Denmark, Djibouti, Dominica, Dominican Republic, East Timor, Ecuador, Egypt, El Salvador, Equatorial Guinea, Eritrea, Estonia, Ethiopia, Faroe Islands, Federated States of Micronesia, Fiji, Finland, France, Gabon, Gambia, Georgia, Germany, Ghana, Greece, Greenland, Grenada, Guam, Guatemala, Guinea, Guinea-Bissau, Guyana, Haiti, Honduras, Hungary, Iceland, India, Indonesia, Iran, Iraq, Ireland, Israel, Italy, Ivory Coast, Jamaica, Japan, Jordan, Kazakhstan, Kenya, Kiribati, Kosovo, Kuwait, Kyrgyzstan, Laos, Latvia, Lebanon, Lesotho, Liberia, Libya, Liechtenstein, Lithuania, Luxembourg, Macedonia, Madagascar, Malawi, Malaysia, Maldives, Mali, Malta, Marshall Islands, Mauritania, Mauritius, Mexico, Moldova, Monaco, Mongolia, Montenegro, Morocco, Mozambique, Myanmar, Namibia, Nauru, Nepal, Netherlands, New Zealand, Nicaragua, Niger, Nigeria, North Korea, Northern Cyprus, Northern Mariana Islands, Norway, Oman, Pakistan, Palau, Panama, Papua New Guinea, Paraguay, Peru, Philippines, Poland, Portugal, Puerto Rico, Qatar, Republic of Congo, Republic of Serbia, Romania, Russia, Rwanda, Saint Kitts and Nevis, Saint Lucia, Saint Vincent and the Grenadines, Samoa, San Marino, Sao Tome and Principe, Saudi Arabia, Senegal, Seychelles, Sierra Leone, Singapore, Slovakia, Slovenia, Solomon Islands, Somalia, Somaliland, South Africa, South Korea, South Sudan, Spain, Sri Lanka, Sudan, Suriname, Swaziland, Sweden, Switzerland, Syria, Taiwan, Tajikistan, Thailand, The Bahamas, Togo, Tonga, Trinidad and Tobago, Tunisia, Turkey, Turkmenistan, Tuvalu, Uganda, Ukraine, United Arab Emirates, United Kingdom, United Republic of Tanzania, United States of America, Uruguay, Uzbekistan, Vanuatu, Vatican, Venezuela, Vietnam, Western Sahara, Yemen, Zambia, Zimbabwe. |

* approved, but not necessarily marketed

*Sources: drugs.com (Dec 2024)*

**Supplement data 15.** Longitudinal MRI follow-up of intracranial meningioma in three clinical scenarios.

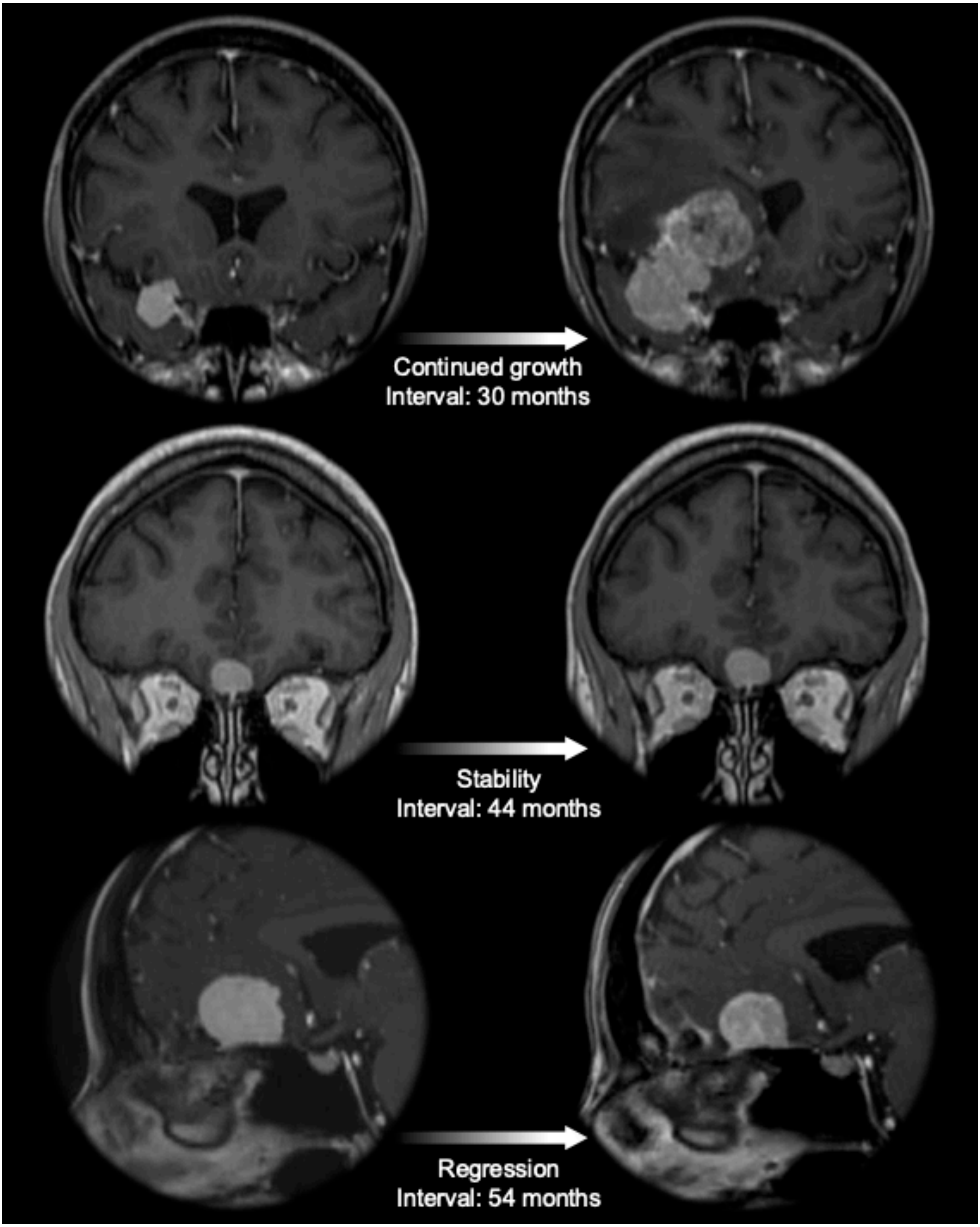

Top row (progression of meningioma under cyproterone acetate treatment). A 22-year-old female patient was incidentally diagnosed with a sphenoidal ridge meningioma in 2011. She had been receiving CPA at a dose of 50 mg daily, continuously, for five years in the context of endometriosis. Following the tumor discovery, the patient was lost of follow-up and continued CPA despite against medical advice. Thirty months later, she returned with symptoms of global psychomotor slowing. Brain MRI revealed a significant increase in tumor volume (from 1.5 $cm^3$ to 39.7 $cm^3$). She underwent emergency surgery due to signs of intracranial hypertension, and CPA was definitively discontinued. At 7-year follow-up, no tumor recurrence was observed.

Middle row (stability of meningioma post-discontinuation of cyproterone acetate). A 43-year-old woman was incidentally diagnosed with a jugum meningioma in 2019. She had been receiving CPA, at a dose of 50 mg daily, 21 days per month, for 23 years due to endometriosis. A conservative approach was adopted, involving regular MRI monitoring every 3 to 6 months and definitive cessation of progestin therapy. At 44 months of follow-up, imaging demonstrated overall stability of the meningioma lesions.

Bottom row (Regression of meningioma following the cessation of cyproterone acetate). A 42-year-old female was incidentally diagnosed with a right anterior clinoid meningioma in 2017. She had been receiving CPA, at a dose of 50 mg daily, continuously, for approximately 10 years. A conservative approach was adopted, involving regular MRI monitoring every 3 to 6 months and definitive cessation of progestin therapy. At 54-month follow-up, imaging showed a significant reduction in tumor volume (from 3.2 $cm^3$ to 1.8 $cm^3$).

*All images shown are derived from MRI examinations performed at GHU-Paris Psychiatrie et Neurosciences, Sainte-Anne Hospital (F-75014 Paris, France). For the upper row, MRI was performed at inclusion on June 28, 2011, and at follow-up on December 30, 2013; for the middle row, at inclusion on January 6, 2020, and at follow-up on September 9, 2024; and for the bottom row, at inclusion on October 21, 2017, and at follow-up on May 27, 2024. The images are fully anonymised, as required under French legislation and approved by the IRB. We confirm that the study was approved by the institutional review board (IRB#1:2024/53; N°IRB00011687). Informed consent was waived for this retrospective observational study in accordance with French regulations.*

**Supplement data 16.** Tumor location

| Study | Anterior skull base | | Medial skull base | | Convexity | |
|---|---|---|---|---|---|---|
| | N | Risk | N | Risk | N | Risk |
| *Hoisnard et al. (2022)* | 779 (31.2%) | OR 10.2 (8.9–11.6) | 757 (30.5%) | OR 9.7 (8.6–11.1) | 698 (28.2%) | OR 5.1 (4.6–5.7) |
| *Roland et al. (2024)* | 37 (25.3%) | RR 3.6 (2.0–6.2) | 47 (32.2%) | RR 4.5 (2.6–7.8) | 42 (28.8%) | RR 2.9 (1.6-4.2) |
| *Weil et al. (2021)* | 190 (36.8%) | HR 47.1 (14.9-149.1) | 130 (25.2) | HR 25.1 (9.1-69.3) | 107 (20.7) | HR 32.5 (11.9-89.1) |
| *Roland et al. (2024)* | 53 (20,7%) | OR 35.28 (25.99-47.88) | 50 (19,5%) | OR 31.21 (22.70-42.90) | 93 (36,3%) | OR 14.86 (11.55-19.10) |
| *Epi-Phare (2024)* | 82 (28,6%) | OR 1,50 (1,17-1,93) | 79 (27,5%) | OR 1,90 (1,47-2,46) | 76 (26,5%) | OR 0,92 (0,72-1,18) |
