# Supplementary material for: Progestogen use and the risk of intracranial meningioma: a systematic review and meta-analysis
Source: eClinicalMedicine. 2026 Feb 12;92:103791. doi: 10.1016/j.eclinm.2026.103791 (PMC12925135; doi:10.1016/j.eclinm.2026.103791)
Supplement: Summary_French [file mmc2.docx]

*The following translations in French were submitted by the authors and we reproduce them as supplied. They have not been peer reviewed. Our editorial processes have only been applied to the original abstract in English, which should serve as reference for this manuscript.*

**Résumé**

*Contexte.* Les méningiomes sont les tumeurs primitives intracrâniennes les plus fréquentes chez l’adulte. Un lien potentiel entre l’exposition aux progestatifs et la survenue de méningiomes intracrâniens suscite des préoccupations légitimes. Nous avons évalué l’association entre différents progestatifs et le risque de méningiome intracrânien, ainsi que des critères secondaires (malignité, localisation, régression).

*Méthodes.* Nous avons mené une revue systématique et une méta-analyse des études épidémiologiques (anglais/français) rapportant une association entre exposition à un progestatif et méningiome intracrânien. Les bases PubMed/MEDLINE, Embase, Cochrane Library et EPI-PHARE ont été interrogées depuis l’origine jusqu’au 1^er^ novembre 2025, complétées par les rapports de pharmacovigilance et une recherche par références. Ont été exclus : travaux non originaux, résumés sans texte intégral, et études sans exposition éligible ou sans critère de jugement pertinent. Le risque de biais a été évalué par l’échelle de Newcastle-Ottawa et la certitude des preuves par GRADE. Des modèles à effets aléatoires ont été utilisés ; l’hétérogénéité a été estimée par I² ; une synthèse narrative a complété l’analyse.

*Résultats.* Parmi 542 références dépistées, 78 études ont été incluses dans la revue et 14 études observationnelles de haute qualité dans la méta-analyse. L’acétate de cyprotérone était fortement associé à une augmentation du risque de méningiome (5 études ; 1047 cas exposés ; OR combiné 12,36 (IC95 % 7,47-20,45) ; I² 73,8 % ; GRADE modéré). L’acétate de médroxyprogestérone dépôt était également associé (6 études ; 842 cas exposés ; OR 2,68 (1,72-4,19) ; I² 92,7 % ; GRADE faible). Des signaux de risque accru étaient rapportés pour l’acétate de chlormadinone, l’acétate de nomégestrol, la promégestone, la médrogestone et le désogestrel. Une régression après arrêt était décrite pour l’acétate de cyprotérone et le nomégestrol. Les tumeurs étaient majoritairement situées à la base du crâne antérieure/moyenne, et les formes malignes semblaient plus fréquentes avec l’acétate de cyprotérone, l’acétate de chlormadinone et l’acétate de nomégestrol.

*Interprétation.* Malgré des limites liées au caractère observationnel, à une confusion résiduelle, à l’hétérogénéité et à l’imprécision pour certaines expositions, certains progestatifs, en particulier les macroprogestatifs à fortes doses, pourraient augmenter le risque de méningiome intracrânien. Une information claire des patients et un suivi clinique, et radiologique si indiqué, sont essentiels.

*Financement.* Aucun.
